# MAPPING current decision-making pathways and reimbursement processes for high-risk medical devices in EU/EEA member states and the UK: a scoping review

**DOI:** 10.1017/S026646232510319X

**Published:** 2025-10-20

**Authors:** Rasha A. Alshaikh, Kieran A. Walsh, Fatma El-Komy, Susan Spillane, Marie Carrigan, Louise Larkin, Patricia Harrington, Michelle O’Neill, Conor Teljeur, Máirín Ryan, Caitriona M. O’Driscoll

**Affiliations:** 1 School of Pharmacy, University College Cork, Cork, Ireland; 2 Faculty of Pharmacy, Tanta University, Tanta, Egypt; 3 Health Technology Assessment Directorate, Health Information and Quality Authority, Cork, Ireland; 4 Department of Pharmacology & Therapeutics, Trinity College Dublin, Trinity Health Sciences, Dublin, Ireland

**Keywords:** health technology assessment, in vitro diagnostics, reimbursement, joint clinical assessment, EU health policy

## Abstract

**Objectives:**

The reimbursement of, and subsequent patient access to, high-risk medical devices (MD) and in vitro diagnostics (IVD) across Europe often vary. The Health Technology Assessment Regulation (HTAR) aims to standardize clinical evaluations through Joint Clinical Assessments. Still, national differences in reimbursement frameworks and evidence integration for MD/IVD may impede the realization of HTAR’s expected benefits. This review aims to map existing reimbursement frameworks for high-risk MD/IVD, identify key oversight structures, and evaluate the use of comparative effectiveness and safety evidence in reimbursement decisions across the EU/EEA/UK.

**Methods:**

A scoping review was conducted according to the registered protocol (osf.io/65bdk) and was reported following the PRISMA-ScR guidelines. Results were validated through direct engagement with national organizations.

**Results:**

Reimbursement frameworks across the EU/EEA/UK for MD/IVD vary significantly. Of the thirty-four countries reviewed, twenty-three incorporate HTA for MD/IVD reimbursement decisions; of these, only eleven countries have a formal HTA process as part of reimbursement pathways. Eight countries have structured mechanisms to address safety and effectiveness evidence uncertainty. Furthermore, twelve countries have primarily centralized processes, while six rely on regional or local decision-making.

**Conclusions:**

This review highlights the variations in how countries integrate HTA into reimbursement frameworks for MD/IVD, how the national decisions are implemented, and how the evidence uncertainty is assessed. Some countries have a well-established reimbursement framework with formal HTA components, whereas others rely on ad hoc HTA processes. Understanding these differences can help optimize the use of HTAR-generated evidence. Further research is needed to capture ongoing reforms in response to the HTAR.

## Background

In Europe, the regulatory landscape for medical devices (MD) and in vitro diagnostics (IVD) is structured quite differently from that of medicinal products, and it differs from the regulatory model used by the US Food and Drug Administration (FDA) for medical devices. There is currently no comparable centralized body responsible for the assessment of all medical devices, like that provided by the European Medicines Agency (EMA) for medicinal products (1). Regulation of MD/IVD in Europe is governed by a system of Notified Bodies and conformity assessment. Notified bodies are independent designated organizations supervised by the member state in which they are located, and they serve to examine the evidence of medical device performance and production procedures to conclude on the safety, effectiveness, benefit, and risk of medical devices, among other factors (2). Compliant devices receive the Conformité Européenne (CE) mark, indicating that the device meets the specified safety, health, and environmental requirements for its category as dictated by the EU regulation (1;3). However, unlike the traditional expectation of randomized controlled trials (RCTs) for the assessment of medicinal products, MD development in Europe has not historically centered on RCTs; this has resulted in patient safety concerns, particularly for high-risk MD/IVD (4).

High-profile adverse events such as the Poly Implant Prothèse (PIP) breast implant scandal (5) and complications associated with transvaginal mesh (6) further exposed weaknesses in earlier regulatory frameworks, underscoring the need for more stringent evidence requirements and postmarket surveillance mechanisms. These incidents triggered regulatory reforms, including the introduction of the Medical Device Regulation (MDR) (EU regulation 2017/745) (7) and the in vitro Diagnostic Regulation (IVDR) (EU 2017/746) (3;8). The MDR and IVDR, which took full effect in May 2021, categorize MD and IVD according to their risk (MDR categorizes medical devices into Class I (low risk), Class IIa (moderate risk), and Classes IIb and III (high risk). IVDR categorizes IVD into Class A (lowest risk), Class B (low to moderate risk), Class C (moderate to high risk), and Class D (highest risk for individuals and public health), and also introduce particular requirements for thorough clinical investigations of high-risk devices (9).

Patient access requires a public or private process of reimbursement or procurement of the device. Reimbursement for medical devices can be defined as a financial payment from a public or private insurer, often directed to a healthcare provider, to cover expenses incurred when using a medical device or conducting a specific procedure (10). To secure reimbursement, payers usually require evidence that the technology improves patient outcomes with acceptable cost-effectiveness. Such evidence is frequently assessed through a national Health Technology Assessment (HTA) process, which considers the device’s added clinical and economic benefits relative to current usual care.

The clinical evidence required by the MDR is now complemented by the Health Technology Assessment Regulation (HTAR) (EU 2021/2282), which came into force on 11 January 2022, and applies from 12 January 2025 (11). HTAR establishes a framework for Joint Clinical Assessment (JCA), aiming to unify comparative clinical effectiveness and safety assessment of high-risk MD and IVD, promote consistent methodology, reduce duplication, and facilitate faster patient access to these technologies (12). HTAR Implementation will take place in gradual phases, starting with oncology medicines and advanced therapy medicinal products in 2025, high-risk MD/IVD in 2026, and orphan medicines in 2028, with all new medicines within scope by 2030 (13). Each country retains authority over its national reimbursement decisions, considering other factors such as healthcare priorities, budgetary implications, and cost-effectiveness (12). As the MDR, IVDR, and HTAR reshape the assessment landscape for MD/IVD, there is a pressing need to understand how these new regulations interact with existing HTA and reimbursement pathways across Europe.

There are limited published reviews that describe the diversity of reimbursement pathways, oversight structures involved, and criteria for patient access to high-risk medical devices in Europe. In particular, there remains limited comparative analysis of how national systems consider safety and effectiveness evidence in guiding reimbursement decisions. Previous reviews have explored assessment and reimbursement frameworks specifically for Companion Diagnostics (14) and digital health technologies (15); however, to the best of our knowledge, none have focused on high-risk MD/IVD.

Therefore, this scoping review aims to provide a comprehensive understanding of the current decision-making frameworks and the key oversight structures governing the reimbursement of high-risk MD/IVD across the EU/EEA/UK just prior to initiation of JCAs for these technologies in 2026. The review also specifically aims to summarize each country’s approach to considering the evidence of comparative effectiveness and safety in the reimbursement decision, highlighting unique elements within each jurisdiction’s pathway.

## Methods

A scoping review was undertaken to explore the current reimbursement policy landscape and identify gaps in information. The reporting of this scoping review follows the Preferred Reporting Items for Systematic Reviews and Meta-Analyses extension for Scoping Reviews (PRISMA-ScR) guidelines (16). The protocol was developed in alignment with the PRISMA-P statement and has been informed by methodologies outlined by Arksey and O’Malley (17), Levac et al. (18), and the Joanna Briggs Institute (19). The protocol was registered in the Open Science Framework (OSF) database in March 2025 (20). There were no notable deviations from the planned protocol.

### Research questions and objectives

The primary aim of this scoping review was to answer the following research questions (RQ):

***RQ 1:** What are the key pathways, stages of decision-making, and oversight structures involved in the reimbursement of high-risk medical devices and diagnostics in the EU/EEA and the UK?*

***RQ 2:** How are comparative effectiveness and safety data for high-risk medical devices and diagnostics used in reimbursement decisions in the EU/EEA and the UK?*

The research questions were developed using the PCC (Population, Concept, Context) framework (Supplementary Table S1), guiding the identification and selection of publications relevant to these specific areas as described in Supplementary Table S1 and the published protocol (20).

The focus of this scoping review is to explore the current reimbursement frameworks, oversight structures involved, and the role of HTA in reimbursement pathways of high-risk MD/IVD, and identify information gaps. For the purpose of this review, HTA can be defined as “*a multidisciplinary process that uses explicit methods to determine the value of a health technology at different points in its lifecycle. The purpose is to inform decision-making in order to promote an equitable, efficient, and high-quality health system*” (21). MD is defined under Regulation (EU) 2017/745 as *“any instrument, apparatus, appliance, software, implant, reagent, material or other article intended by the manufacturer to be used, alone or in combination, for human beings for one or more specific medical purposes specified in the Regulation*” (7). IVD is defined under Regulation (EU) 2017/746 as *“any medical device which is a reagent, reagent product, calibrator, control material, kit, instrument, apparatus, piece of equipment, software or system, whether used alone or in combination, intended by the manufacturer to be used in vitro for the examination of specimens, including blood and tissue donations, derived from the human body, solely or principally for the purposes specified in the Regulation”* (8).

### Search strategy and identification of relevant records

Data for this scoping review were extracted from organizational websites, bibliographic databases, and grey literature sources, as described below. Of note, given the broad and sometimes inconsistent nature of the different information sources, the search process was iterative and often required the research team to cycle between different sources to establish a full and accurate picture of the national reimbursement pathway. No language restrictions were applied. DeepL Pro or Google Translate was used for translation for non-English language sources. Only records since 2017 were included in the review, which corresponds to the period when the MDR and IVDR came into effect (20).

### Organizations search

The websites of key organizations involved in HTA and policymaking were searched between August and November 2024, as listed in Supplementary Table S2 (Supplementary data) (20). These organizations represent key HTA and reimbursement decision-making institutions in EU/EEA countries and the UK.

### Database search

The following databases were searched on 3 September 2024: Medline Complete (via EBSCOhost, Supplementary Table S3), Embase (via Elsevier, Supplementary Table S4), Cochrane Library (via Wiley, Supplementary Table S5), INAHTA Database (Supplementary Table S6); a summary of the results obtained in each search is presented in Supplementary Table S7. The Medline Complete search was rerun on 20 January 2025.

### Grey literature search

Google Scholar was searched between September and November 2024 for relevant sources. Furthermore, selected HTA-focused journals, namely Value in Health and the International Journal of Health Care and Technology, were searched manually to identify relevant articles. Also, a Google search was conducted in November 2024; the search was limited to the first three pages of results obtained.

### Screening, data extraction, and data analysis process

**Figure 1.**
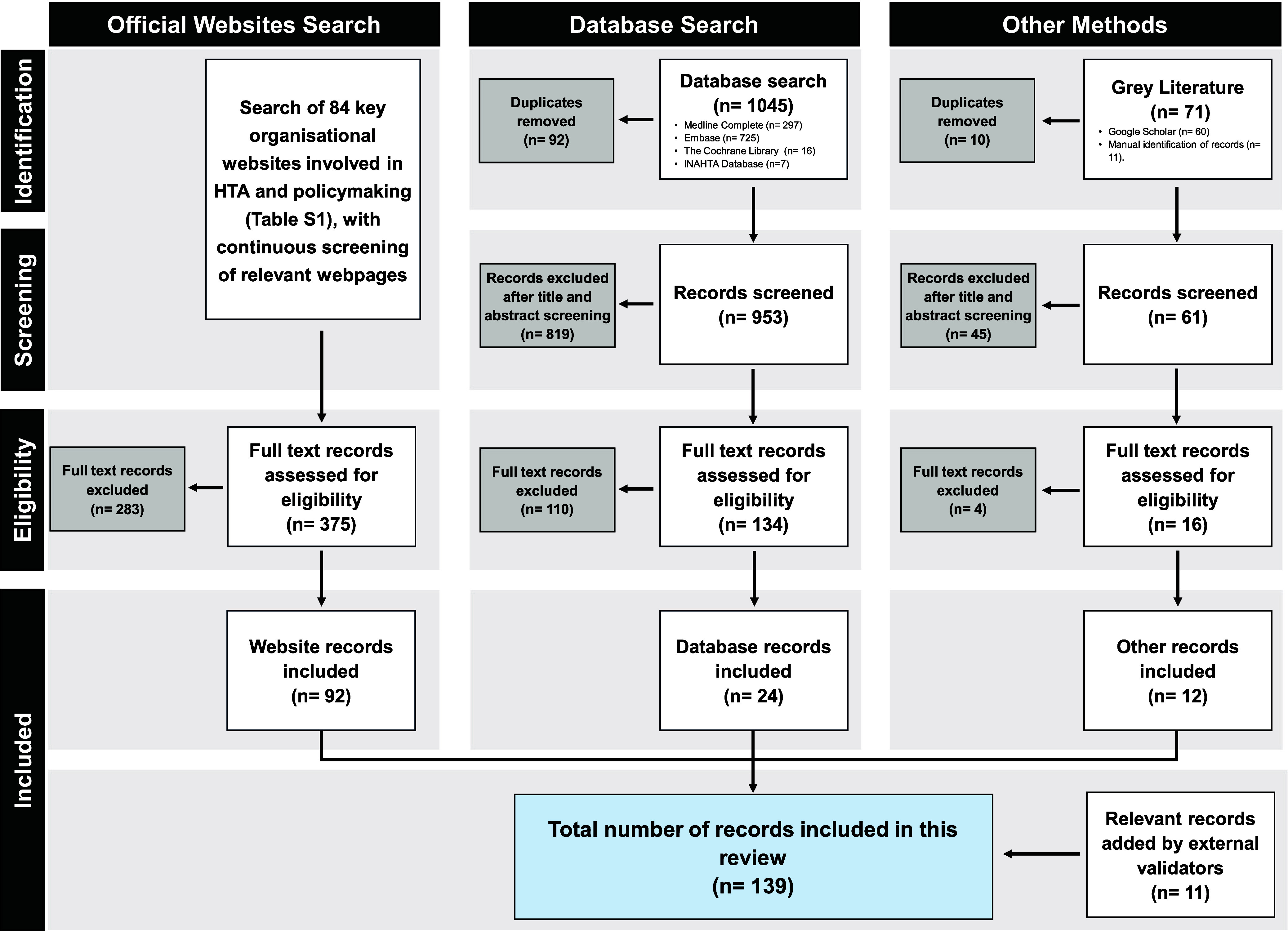
PRISMA-ScR flow diagram of the records identification and screening process for the scoping review.

All identified citations were imported into EndNote, and duplicates were systematically removed. The citations were then imported into Covidence, and two reviewers independently screened the titles, abstracts, and full texts to determine relevance based on the predefined inclusion and exclusion criteria, with a third reviewer consulted for disagreements (20). The complete selection process is detailed in the PRISMA-ScR flow diagram (Figure 1).

A standardized data extraction template for this review was developed in Microsoft Excel (Supplementary Table S8) and was used to capture all essential information via specific extraction questions (20). Data extraction was conducted by one reviewer (Alshaikh RA), and each entry was independently cross-checked by a second reviewer (El-Komy FW) to ensure all relevant information was accurately captured. Any differences in interpretation were discussed to reach a consensus, with any disagreements resolved by discussion with a third reviewer (Walsh KA). Consistent with established scoping review methodology, this study did not conduct a formal quality appraisal of included publications (19).

### Data validation

To confirm that the findings of this review accurately reflect the current national practices in reimbursing high-risk MD/IVD, a validation process was conducted between December 2024 and January 2025. Representatives from HTA organizations, ministries of health, or other relevant reimbursement decision-making bodies were directly contacted (via email) and asked to review and provide feedback on the national information. A list of organizations that provided data validation is presented in Supplementary Table S9. Any additional information, comments, and modifications provided by the external organization were incorporated into this report.

## Results

### Publication selection

The records included in this review were sourced from organizational websites, databases, and grey literature. The search of organizational websites listed in Supplementary Table S2 resulted in the retrieval of 375 unique records. Of these, only 92 records were relevant and included in the study, and 283 records were excluded after full-text assessment (Figure 1).

For the database search, a total of 1,045 records were identified (details of search results are described in Supplementary Table S7), comprising 953 unique publications and 92 duplicates. A total of 819 publications were excluded based on the title and abstract screening, and the full texts of the remaining 134 records were assessed for eligibility, resulting in the inclusion of twenty-four publications in the study (Figure 1). The agreement rate between the first and second reviewers was approximately 98 percent, which was considered acceptable. The Medline Complete search was rerun on 20 January 2025, and three additional results were identified, which were excluded based on title and abstract screening. Finally, the grey literature search resulted in the inclusion of twelve records (Figure 1). References are cited in their respective location in the manuscript and Supplementary Materials.

A comprehensive summary of the information for each country was sent for external validation to national organizations in thirty-four countries. Feedback was received from thirty of thirty-four contacted countries (88 percent response rate, with England, Scotland, Wales, and Northern Ireland counted separately). The organizations that provided feedback or verified the information are listed in Supplementary Table S8. This validation process led to the inclusion of 11 additional records (publications, official website pages, and documents) provided by the external validators. As a result, a total of 139 records were incorporated into the study (Figure 1).

### Implementation of Health Technology Assessment for the medical devices process across the EU/EEA/UK

This study examines the role of HTA in different reimbursement pathways of high-risk MD and IVD across the EU, the EEA, and the UK.

**Figure 2.**
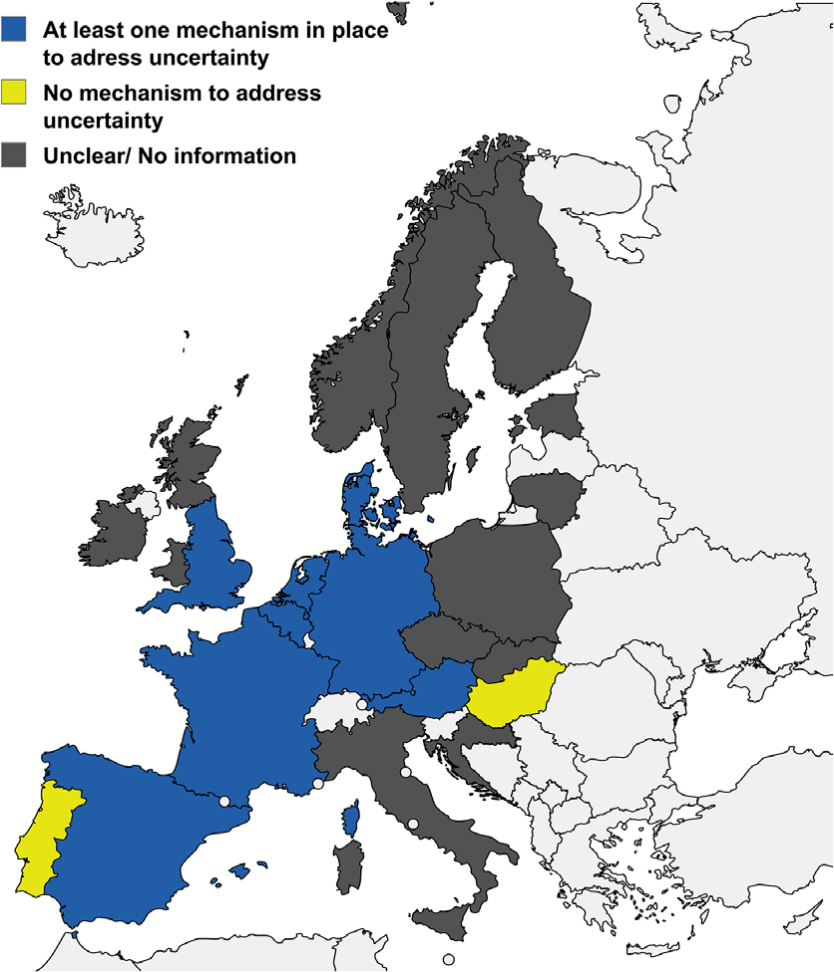
Formal mechanisms to address evidence uncertainty in the reimbursement of high-risk MD/IVD across countries with any form of HTA for medical devices (as of November 2024). Countries are categorized according to the presence or absence of mechanisms to address the uncertainty of comparative safety and effectiveness evidence within the reimbursement frameworks. Only countries with reported forms of HTA for reimbursement of medical devices are described in this figure (*n* = 23 countries). The figure was created using mapchart.net.

Specifically, the analysis includes all twenty-seven EU member states, Iceland, Liechtenstein, Norway, and the UK (Figure 2), totaling thirty-four countries (with England, Scotland, Northern Ireland, and Wales considered separately). The results show that HTA for high-risk MD/IVD is not established in all EU/EEA/UK countries. Of the thirty-four countries in this review, we identified publicly available information on the reimbursement process for high-risk MD/IVD in thirty-one countries; there was no detailed public information on MD/IVD reimbursement pathways for three countries (Luxembourg, Liechtenstein, and Iceland).

Of the thirty-one countries with publicly available information, any form of HTA process for medical devices (including high-risk MD/IVD) was reported in the reimbursement decision-making process in twenty-three countries (Table 1). Eight countries confirmed the lack of an established HTA process for medical devices, and no information was found regarding HTA and reimbursement decision-making in the remaining three countries (Table 1). Due to information scarcity, countries without a reported HTA process for high-risk MD/VD (n = 8) and those without publicly available information on HTA processes (n = 3) are excluded from further analysis in this review.

**Table 1.** Reported HTA processes for reimbursement decisions of medical devices across the EU/EEA/UK as of November 2024

Category^a^	Number	Countries
Countries with any form of HTA for high-risk MD/IVD.	23 countries	Austria, Belgium, Croatia, Czech Republic, Denmark, Estonia, Finland, France, Germany, Hungary, Ireland, Italy, Lithuania, Netherlands, Poland, Portugal, Slovakia, Spain, Sweden, England, Scotland, Wales, Norway.
Countries with no form of HTA for high-risk MD/IVD.	8 countries	Bulgaria, Cyprus, Malta, Latvia, Greece, Romania, Northern Ireland, Slovenia.
Countries with no public information on HTA processes for high-risk MD/IVD.	3 countries	Luxembourg, Liechtenstein, Iceland.

a The information for 30 countries (88 percent) was verified by national organizations listed in Supplementary Table S9.

### Key oversight structures involved in decision-making on patient access to medical devices in the EU/EEA/UK

For the twenty-three countries with any form of HTA for medical devices, the process summary and oversight structures involved in assessment and reimbursement decision-making are detailed in Table 2. Furthermore, oversight structures involved in the initiation and prioritization of reimbursement requests are described in Supplementary Table S10. Generally, in these countries, the reimbursement process for high-risk MD/IVD is organized in a structured, stepwise approach with four main stages. The first stage is the topic identification, with or without a prioritization step (Supplementary Table S10). The second step is the assessment (i.e., HTA) stage, where the evidence of comparative safety and effectiveness of the device, among other factors, is assessed to generate an output (e.g., recommendation, advice, or conclusion) (Table 2). In this stage, there is considerable variability in how the assessment is done and how the evidence is considered. Some countries rely on evidence synthesis, appraisal of manufacturer submissions, or both. The specifics of the assessment stage are described in detail in a published European network for the HTA (EUnetHTA) report (22). Additionally, the scope of HTA varies across countries in terms of whether it is limited to comparative clinical effectiveness and safety or extends to other domains such as economic evaluation, organizational impact, and ethical considerations. These details are thoroughly described in Supplementary Table S11.

**Table 2.** Overview of the oversight structures involved in the assessment and reimbursement of high-risk MD/IVD across the EU/EEA/UK as of November 2024

Country (reimbursement pathway)		Assessment (HTA)	Post-HTA decision-making^a^		Funding/reimbursement decision	Ref.
Austria (Applications for new reimbursement groups under LKF).		Selected interventions undergo STA/MTA by **AIHTA**.	The assessment **by AIHTA** includes scientific conclusions with recommendations.		Reimbursement decisions are made by the **Austrian FHC**. Purchasing decisions are handled at the hospital level or by centralized public hospital cooperatives in the federal states.	(23;24)
Belgium		The **CTIIMH-CRIDMI** appraises the submission and formulates a provisional reimbursement proposal. In this stage, **KCE** scientific advice might be considered. However, MD reimbursement is not legally bound to **KCE** recommendations.	Manufacturer input is invited on the generated proposal in the assessment stage. Then, **CTIIMH-CRIDMI** formulates the final reimbursement proposal.		The **Minister of Social Affairs and Public Health** finalizes the decision, pending budgetary approval **from the Minister of Budget**.	(25–31)
		**Internal evaluators** produce an evaluation report (HTA).	After the manufacturer’s input, **CTM’s Clinical Biology working group** discusses and validates the evaluation report, generating a reimbursement proposal.		The approved reimbursement proposal is forwarded for decision to the **Minister of Social Affairs and Public Health** and **the Council of State**.	
Croatia		**CHIF** appraises manufacturer submissions for reimbursement decisions. In certain cases, HTA (performed **by Croatian MoH**)^b^ might be requested by **CHIF**.	The assessments **by the Croatian MoH** include scientific conclusions and recommendations.		The final decision is made by **Croatian decision-makers**.	(32–34)
Czech Republic		Assessed and categorized by the **Public Health Insurance.**	Unclear		The **Public Health Insurance** manages the reimbursement.	(35–38)
Denmark		The HTA is performed **by the DHTC.**	The assessments by **DHTC** include scientific conclusions and recommendations.		The **regional governments’ procurement organizations** and **the Regions Joint Procurement** are responsible for the procurement.	(39;40)
Estonia		**EHIF** assesses all requests for medical device reimbursement. In certain cases, the **University of Tartu HTA Centre** prepares HTA reports, with topics selected by the **Estonian HTA Expert Council** based on their relevance to national healthcare priorities.	**EHIF** assessments will conclude with a decision on reimbursement. **The University of Tartu HTA Centre** produces evaluation reports with a recommendation.		**EHIF** is responsible for funding and managing reimbursement processes.	(41;42)
Finland		**COHERE Finland** primarily conducts national-level HTA. **FinCCHTA** primarily conducts hospital-level HTA.	The assessments **by FinCCHTA or COHERE Finland** include scientific conclusions and recommendations.		Reimbursement is undertaken by **Kela**.	(43–45)
France		**CNEDiMTS** (one of the specialized Committees of HAS) performs national-level HTA. **CEESP** (specialized Committees at HAS) conducts health economic assessments for specific devices.	The assessments **by CNEDiMTS** include scientific conclusions and recommendations.		The device tariff is negotiated between the **CEPS** and the developer for devices with favorable reimbursement recommendations. Then, the **Minister of Health** makes the final reimbursement decision based on the opinions of the **CNEDiMTS** and the **CEPS.**	(46–50)
Germany (for high-risk devices with a novel mechanism of action)		**G-BA** commissions the **IQWiG** to perform the HTA.	The assessments by **IQWiG** include scientific conclusions and recommendations.		The **G-BA** makes the final reimbursement decisions under the German **SHI** system.	(51–57)
Hungary		**NEAK** requests a critical appraisal from **TéF** (a department of **NNGYK** responsible for HTA). The opinion of the **Health Professional College** is also requested.	**TéF** appraisals include scientific conclusions and recommendations; together with the **Health Professional College opinion,** they constitute the basis for reimbursement recommendations by **NEAK.**		Submissions with favorable reimbursement recommendations are forwarded to the **Ministry of Interior** for final decision.	(58–60)
Ireland		**HIQA** may conduct HTA of MD/IVD, usually as a component of an innovative national programme.	HIQA HTA include advice to the Minister for Health and the HSE.		Decisions to fund innovative national programs are made by the Minister for Health, having considered the HTA advice.	(61;62)
Italy		**AGENAS** may conduct the HTAs of medical devices.	Unclear		Unclear	(63–65)
Lithuania		**VASPVT** conducts the HTA and then submits a report on the assessment to the **HTA Committee** for consideration.	The **HTA Committee** reviews HTA results and makes recommendations on reimbursement. The recommendation is submitted to the **Minister of Health**.		The **Minister of Health** evaluates recommendations and forwards decisions for reimbursement to the national health insurance fund **(VLK)**. **VLK i**mplements reimbursement decisions based on approvals.	(66–68)
Netherlands		HTA is conducted by **ZIN**.	The assessments by **ZIN** include a final evaluation report with advice on the reimbursement.		The advice by **ZIN** is directed to the **Minister of Health, Welfare, and Sport** on whether to include or exclude medical devices in the basic package.	(69–74)
Poland		The **MoH** may commission **AOTmiT** to appraise the manufacturer’s data using “verification analysis.” **AOTMiT can also perform HTA** in certain conditions. The verification analysis by **AOTmiT** includes a scientific conclusion. A **Transparency Council** reviews the verification analysis and issues a position.	The recommendation is made by **the President of AOTMiT** based on the verification analysis, the Transparency Council’s position, and the original application.		The recommendation is forwarded to the **Minister of Health,** who issues the final action on reimbursement.	(75–79)
Portugal		For valid requests, a technical opinion (clinical evaluation) is requested from **CATS**, a specialized unit in **INFARMED**. **The CATS** report will include a conclusion regarding the technology’s value relative to the comparator. Only devices with equivalent or added value compared to the defined comparators can proceed to the economic evaluation.	Economic evaluation and price negotiation are conducted by **INFARMED**.		The **Ministry of Health** is responsible for approving reimbursement recommendations if an agreement is reached in price negotiations.	(80–82)
Slovakia		As of August 2024, **NIHO** is responsible for assessments of medical devices after a request by **MZSR.**	The assessments by **NIHO** include scientific conclusions and recommendations.		Based on **NIHO’s** recommendation, **MZSR** will negotiate reimbursement terms with the registration holder.	(83–86)
Spain (updating the content of the Common Portfolio of Services)		HTA is performed by **RedETS**	The assessments by **RedETS** include scientific conclusions and recommendations.		The **National Health Care System** decides on the inclusion of the technology in the Common Portfolio of Services.	(22;64;87–89)
Sweden (joint cooperation model for national assessment and procurement of medical technologies)		**TLV** is responsible for conducting the HTA of selected devices at the request of the **MTP council.**	**TLV assessments** include scientific conclusions and recommendations. The **MTP Council** decides which technologies should be introduced nationally after an HTA and consultations with clinical experts.		The regions are responsible for securing access to medical technologies in healthcare through procurement.	(90–92)
UK	England (new health technologies)	MD and simple diagnostics are routed to **MTEP. MTEP team** drafts the scopes and commissions an **EAG**.	HTA is performed by **EAG**.	The **Medical Technologies Advisory Committee** issues draft recommendations considering HTA results. After consultation and resolution, guidance is issued.	**NHS England** is responsible for the implementation of **NICE** guidance.	(93–98)
		Complex diagnostics are routed to the **DAP**. **DAP team** drafts scopes and commissions an **EAG**.		The **Diagnostics Advisory Committee** issues draft recommendations considering HTA results. After consultation and resolution, guidance is issued.		
	Scotland	**SHTG** assesses the proposed topics.	**SHTG** assessments include scientific conclusions and recommendations.		**NHS Scotland** is responsible for the implementation of **SHTG** advice.	(99;100)
	Wales	**HTW** assesses topics that meet the criteria to produce an Evidence Appraisal Report (EAR).	The **Appraisal Panel** within the **HTW** assesses the EAR and produces the HTW advice and guidance, which includes scientific conclusions and recommendations.		**NHS Wales** is responsible for the implementation of **HTW** advice.	(101;102)
Norway		All HTAs of medical devices are conducted by **NOMA,** except for mini-HTAs. Mini HTAs are conducted by clinicians and supporting units in hospitals.	HTAs by NOMA only include scientific conclusions and do not include recommendations on implementation. The **decision-making group “Decision Forum”** (which consists of the chief executive officers of the four RHAs)^c^ decides whether the device should be reimbursed.		The devices can be reimbursed by the regional health authorities (specialist health care) or the National Insurance Scheme (primary health care).	(103–106)

**AIHTA:** Austrian Institute for Health Technology Assessment. **STA:** Single Technology Assessment. **MTA:** Multiple Technology Assessment. **CTIIMH-CRIDMI**: The Commission of Reimbursement of Implants and Invasive Medical Devices. **INAMI-RIZIV:** National Institute for Health and Disability Insurance. **KCE**: Belgian Health Care Knowledge Centre. **CTM:** Medical Technical Council. **CHIF:** Croatian Health Insurance Fund. **MoH:** Ministry of Health. **DHTC:** Danish Health Technology Council. **EHIF**: Estonian Health Insurance Fund. **FinCCHTA:** Finnish Coordinating Center for Health Technology Assessment. **COHERE Finland**: Council for Choices in Health Care in Health Care in Finland. **Kela**: Social Insurance Institution of Finland. **CNEDiMTS:** Medical Device and Health Technology Evaluation Committee. **HAS:** French National Authority for Health. **CEPS**: Economic Committee for Health Products. **CEESP:** The Commission for Economic and Public Health Evaluation in France. **HSE:** Health Service Executive. **InEK:** Hospital Remuneration System. **G-BA**: Federal Joint Committee in Germany. **IQWiG**: The Institute for Quality and Efficiency in Healthcare. **SHI:** Statutory Health Insurance in Germany. **FHC:** Federal Healthcare Commission. **NEAK:** National Health Insurance Fund in Hungary. **TéF:** Technology Assessment Department in Hungary. **NNGYK:** National Centre for Public Health and Pharmacy in Hungary. **HIQA:** Health Information and Quality Authority. **AGENAS:** National Agency for Regional Healthcare Services. **SSN**: National Health Service in Italy. **VASPVT**: State Accreditation Service for Health Care Activities under the Ministry of Health of the Republic of Lithuania. **VLK:** National Health Insurance Fund in Lithuania. **ZIN:** the National Health Care Institute in the Netherlands. **AOTMiT:** Agency for Health Technology Assessment and Tarification. **INFARMED:** the National Authority for Medicaments and Health Products. **CATS:** The Portuguese Commission for Health Technology Assessment. **NIHO:** National Institute For Value And Technologies In Healthcare. **MZSR:** The Ministry of Health of the Slovak Republic. **RedETS:** Spanish Network of Health Technology Assessment Agencies. **TLV:** The Dental and Pharmaceutical Benefits Agency. **MTP Council**: Medical Technology Product Council in Sweden. **NICE:** National Institute for Health and Care Excellence (England). **MTEP:** Medical Technologies Evaluation Programme (England). **DAP:** Diagnostics Assessment Programme (England). **EAG:** External Assessment Group. **HTW:** Health Technology Wales. **SHTG**: Scottish Health Technologies Group. **NHS**: National Health Service (UK). **NOMA:** Norwegian Medical Products Agency (previously called the Norwegian Medicines Agency).

a “Unclear” was used when some information was available but lacked sufficient detail to describe how prioritization occurs. “No information” was used when no relevant description of prioritization processes could be identified.

b HTA in Croatia was performed by the Agency of Quality and Accreditation in Health Care (the Department for Development, Research, and HTA) from 2009 until 2018, before being taken over by the MoH on 01/01/2019.

c The four regional health authorities (RHAs) are: Central Norway Regional Health Authority (“Helse Midt-Norge”); Northern Norway Regional Health Authority (“Helse Nord”); Southern and Eastern Norway Regional Health Authority (“Helse Sør-Øst”); and Western Norway Regional Health Authority (“Helse Vest”).

The third stage is the recommendation stage, where different outcomes are concluded based on the assessment stage. In some cases, the assessment does not include a formal recommendation; in other cases, a clear recommendation regarding device reimbursement is provided (Table 2). Finally, the fourth and final step is the reimbursement decision, where a decision is made regarding funding. Depending on the national context, this decision may be contingent on the outcome of the HTA assessment, may consider the output in the context of other financial or organizational aspects, or may be made independent of it.

Different stakeholders in each country can initiate the reimbursement process for MD and IV (described in detail in Supplementary Table S10). After proposal submission, a formal prioritization process before assessment was reported in at least eight countries. Then, selected technologies, with or without prioritization, proceed to the assessment stage (Supplementary Table S10). The HTA involves evaluating comparative safety and effectiveness, among other criteria unique to national requirements, as explained in the following section. Depending on the national context, these assessments may rely on an appraisal of manufacturer submissions, incorporate independent evidence synthesis, or combine both approaches.

All twenty-three countries have at least one centralized pathway for conducting HTA. In nineteen countries, assessments are carried out by independent bodies (e.g., CNEDiMTS of HAS in France and IQWiG in Germany). In one country, HTA is conducted directly by the Public Health Insurance body (Public Health Insurance in the Czech Republic). Additionally, three countries (Belgium, Croatia, and Estonia) have two organizations involved in the assessment of MD/IVD for reimbursement purposes. The first organization is primarily responsible for appraising manufacturer-submitted evidence to generate reimbursement recommendations; these are the National Institute for Health and Disability Insurance (RIZIV-INAMI) in Belgium, the Croatian Health Insurance Fund (CHIF) in Croatia, and the Estonian Health Insurance Fund (EHIF) in Estonia. The second organization generally provides an independent evidence synthesis, separate from manufacturer submissions, mainly when the submitted evidence is incomplete. These bodies include the Belgian Health Care Knowledge Centre (KCE) in Belgium, the Ministry of Health in Croatia, and the University of Tartu in Estonia (Table 2).

Generally, the assessment results include a scientific conclusion accompanied by a recommendation. In nineteen of twenty-three countries, the HTA recommendation is forwarded to a separate independent body (distinct from the HTA-performing organization) to develop a final decision or recommendations regarding the reimbursement of the MD/IVD. In contrast, in one country (the Czech Republic), the HTA recommendations are produced by the reimbursement decision-making body itself (the Public Health Insurance body), which directly determines the reimbursement outcomes. In three countries (Belgium, Croatia, and Estonia), both approaches coexist; HTA can either be performed by an independent body and then forwarded to the decision-making authority or be produced directly by the reimbursement decision-making body (RIZIV-INAMI in Belgium, CHIF in Croatia, and EHIF in Estonia). Finally, the conclusions and recommendations of HTA are used, among other factors, to inform the reimbursement of devices by public healthcare providers and public health insurance funders (Table 2).

Countries with no formal HTA processes for high-risk MD/IVD (Bulgaria, Cyprus, Malta, Latvia, Greece, Romania, Slovenia, and Northern Ireland) may rely on central or local procurement processes without significant contribution of HTA to decision-making. This suggests that the reimbursement decisions in these settings may be largely influenced by financial considerations; however, the determination of what informs these decisions was beyond the scope of this review.

### The role of HTA in reimbursement pathways of high-risk MD/IVD

For the twenty-three countries with any form of HTA for high-risk MD/IVD in place, the level to which HTA is integrated into reimbursement frameworks and criteria for HTA assessment varies significantly. The data reflect considerable variability in the extent to which the HTA recommendations influence national reimbursement. The countries can be generally grouped into the following three main categories:

1. Countries where HTA is a formal element of certain reimbursement pathways include Austria, Germany, France, Belgium, Norway, Hungary, Sweden (based on the Swedish Joint Cooperation Model for assessing and procuring medical technology), Spain, Lithuania, Portugal, and England.
2. Countries where HTA is not a formal part of any reimbursement pathway (forms an optional/conditional element), include The Netherlands (open reimbursement system for MD/IVD, allowing reimbursement without ZIN assessment, with ZIN providing HTAs to address disputes between physicians, patients, and insurers regarding the inclusion of certain MD/IVD in health care packages (110))**, Croatia, Estonia, Slovakia, Ireland, Italy, Scotland, Wales.
3. Countries where it is unclear if HTA is a formal part of reimbursement pathways include the Czech Republic, Denmark, Finland, and Poland.

Extended information for each of these countries is provided in Supplementary Table S11, which provides an overview of key national reimbursement mechanisms for high-risk MD/IVD, the role of HTA in each country’s reimbursement process, and factors considered in HTA recommendations and final decisions.

### Mechanisms to address the uncertainty of comparative safety and effectiveness evidence for high-risk MD/IVD

For countries with reported HTA for MD/IVD, there is significant diversity in how each country manages uncertainty in relation to comparative safety and effectiveness evidence. Such uncertainty may originate from clinical, economic, or safety data gaps, which may be incomplete or inconclusive. A summary of how countries deal with evidence uncertainty is presented in Figure 2. The countries can be generally grouped into the following three main categories:

1. Countries with an established formal mechanism for addressing evidence uncertainty (eight countries; Figure 2). For these countries, there is a dedicated reimbursement route in case of evidence uncertainty. The funding mechanisms, reimbursement conditions, and criteria in the context of uncertainty are summarized in Supplementary Table S12. The funding decisions, in this case, are often linked to specific criteria, the most common being the potential added clinical benefit of the device, disease severity, and the device’s ability to address unmet or partially met clinical needs in the national context. In these countries, the devices can be reimbursed under different conditions aimed at addressing this uncertainty, including managed access agreements, reimbursement exclusively in research conditions, initiating clinical trials, and coverage with evidence development.
2. Countries with no formal mechanism to address evidence uncertainty (two countries; Figure 2).
3. Countries with unclear processes or no information regarding the mechanisms for addressing uncertainty, which suggests the lack of a formal process (12 countries; Figure 2). This category also includes countries that might have a specific outcome for HTA in case of evidence uncertainty; however, no complementary mechanism for reimbursement has been identified.

### Regional variations and the level of centralization in the reimbursement framework and decisions implementation

**Figure 3.**
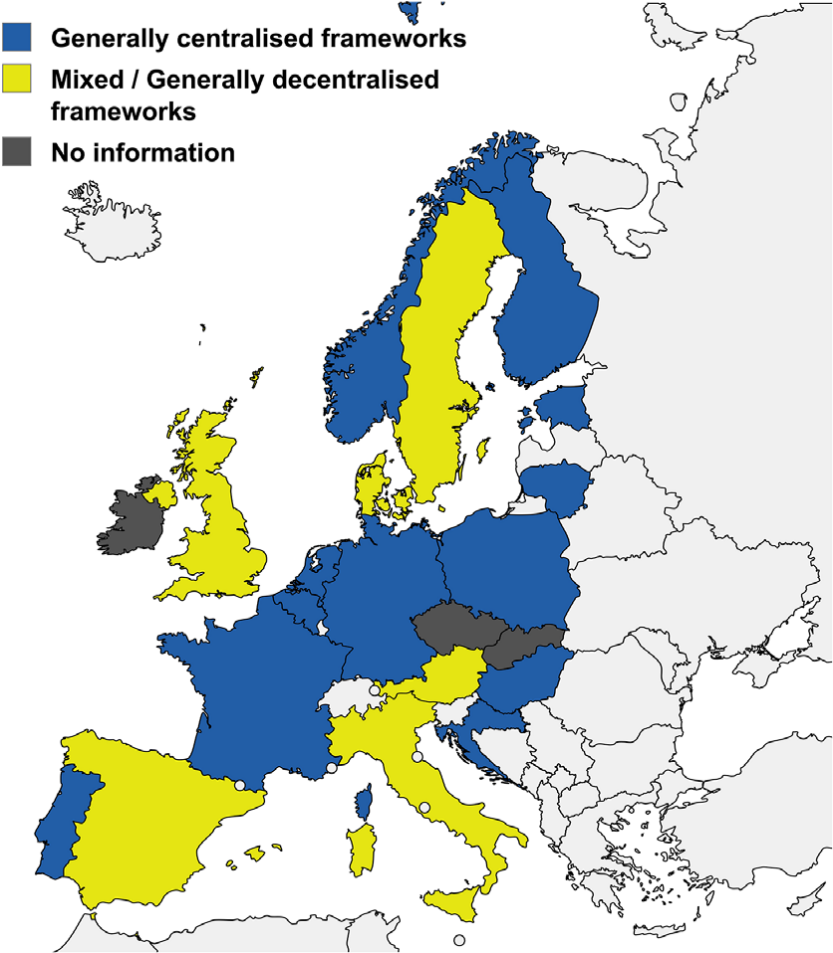
Centralization levels of reimbursement frameworks for MD/IVD across countries with any form of HTA for medical devices (as of November 2024). Countries are categorized according to the extent of national centralization of reimbursement frameworks. Only countries with reported forms of HTA for reimbursement of medical devices are described in this figure (n = 23 countries). The figure was created using mapchart.net.

As previously mentioned, all the countries with any form of HTA for high-risk MD/IVD have at least one mechanism for national-level HTA assessment and generation of recommendations for these devices. However, the locus of authority and decision-making structures governing the reimbursement of high-risk MD/IVD vary significantly. This results in different levels of regional variabilities of medical device reimbursement. The level of centralization in the reimbursement frameworks for each country is summarized in Figure 3 and further described in detail in Supplementary Table S13.

Countries can be generally classified into two main categories. The first includes those countries with generally centralized reimbursement frameworks (12 countries), where the vast majority of reimbursement decisions occur at the national level, with an expected homogeneous reimbursement and implementation throughout the country (Figure 3).

The second category includes countries with mixed or generally decentralized reimbursement frameworks (six countries), where the majority of medical devices are being adopted through regional or hospital-level device uptake, with or without local assessment. While some countries within this category may have a national-level reimbursement scheme, it is typically secondary to regional or local hospital-level processes, resulting in more variability in how medical devices are adopted. Hospital-based HTA activities vary considerably but can include horizon scanning, early assessments, prioritization, and other related aspects of HTA. Regarding its role in hospital-level decision-making, recommendations from hospital-based HTA may be mandatory or optional. (107)

Some of these countries might have limited integration of HTA overall in the reimbursement decisions of high-risk MD/IVD. This category also includes countries with mixed systems, where centralized HTA takes place at the national level; however, reimbursement decisions are still made at the regional or local level. In these cases, national HTA recommendations might provide advice for local decisions, but regional authorities are ultimately responsible for decision-making. No information was found regarding the status of centralization in the three countries (Ireland, Slovakia, and the Czech Republic; Figure 3).

## Discussion

This scoping review highlights the significant variations in the reimbursement frameworks for high-risk MD/IVD across the EU/ EEA/UK. Some countries have well-established, centralized HTA processes that play a crucial role in reimbursement decisions for MD/IVD, whereby a positive reimbursement decision is largely contingent on HTA demonstrating added clinical benefit and, in certain cases, cost-effectiveness. Other countries have more ad-hoc HTA processes or lack formal HTA structures for high-risk MD/IVD. Notably, Western European countries tend to have more established and centralized HTA frameworks and generally make their reimbursement decision-making processes publicly available. Several Eastern European countries and smaller states across the EU/EEA had limited publicly available data, which may reflect limited structured HTA processes and/or resource and capacity constraints. The formalization of HTA frameworks might affect the timeline or uniformity of patient access to high-risk MD/IVD, which represents an important area for future comparative research.

The findings also demonstrate how comparative safety and effectiveness data affect reimbursement decisions. While some countries adopt provisional reimbursement schemes or conditional coverage pathways to cope with uncertainty in the evidence, most countries lack such formal schemes. The introduction of JCA is expected to improve the quality of evidence for high-risk MD/IVD and reduce the level of uncertainty in the evidence informing national reimbursement decisions over time. The structured nature of the assessment scope proposals may indirectly shape industry practices. (108) By requiring manufacturers to address specific research questions relevant to multiple Member States, JCAs could encourage the generation of more relevant and robust evidence.

The findings are particularly relevant given the HTAR mandates that Member States give “due consideration” to JCAs in national decision-making. (109) The Regulation represents a significant shift, aiming to improve consistency, reduce duplication of effort, and ultimately accelerate patient access to high-risk devices with demonstrated added value. These expectations, however, are likely to be realized to varying degrees across Member States according to the degree to which national systems can align with the requirements. Indeed, for countries with developing or yet-to-be-developed HTA processes, JCAs may have limited immediate impact. In these contexts, the absence of a coherent national system into which JCA findings can be meaningfully integrated may impede the realization of the Regulation’s intended benefits.

The results have significant implications for health technology developers, assisting them in understanding the diversity and trajectory of national reimbursement systems, which is essential for aligning evidence-generation strategies with changing requirements due to the implementation of the HTAR. The review also has implications for national HTA agencies, policymakers, clinicians, patients, and payers impacted by the HTAR, as mapping existing systems will guide capacity building, implementation planning, understanding access variability, and potentially enabling cross-country collaboration.

A key strength of this review is its ability to capture the reimbursement pathways and HTA involvement for high-risk MD/IVD across the EU/EEA/UK, most of which will be affected by the HTAR. Also, the versatility of sources used and the high verification rate by the national organizations ensure a reliable portrayal of national processes. However, one notable limitation of this review is the scarcity of information available for many countries. Future qualitative interviews with stakeholders from European HTA bodies, national decision-makers, and policymakers would represent an important area for future research to complement our findings. These studies could enhance our understanding of how countries plan to “give due consideration” to JCA outputs within national reimbursement frameworks. Furthermore, national case studies and participatory research with a wider range of stakeholders (clinicians, payers, industry representatives, and patients) could provide practical insights into how HTA recommendations are operationalized in real-world settings and generate additional perspectives not available through document review alone.

## Conclusion

The introduction of the HTAR presents a unique opportunity for Europe to standardize clinical evaluations, enhance patient safety, and improve access to high-risk MD/IVD. However, the degree to which this opportunity is realized may depend on the ability of individual countries to adjust their national frameworks to take into consideration the JCA reports. This review highlights the significant variations in how EU/EEA/UK countries integrate HTA into national reimbursement frameworks for high-risk MD/IVD, how the national decisions are implemented, and how the uncertainty of evidence is assessed. Some countries have a well-established reimbursement framework with a formal HTA component. Other jurisdictions rely on more ad-hoc HTA processes with reimbursement decisions not necessarily driven by HTA outcomes. Understanding these differences can help optimize the use of HTAR-generated evidence. Further research is needed to capture ongoing reforms and anticipated changes in response to the HTAR, to explore structural gaps, cooperation opportunities between member states, and HTAR implementation challenges, specifically in countries with weak or absent HTA systems.

## Supporting information

Alshaikh et al. supplementary material Alshaikh et al. supplementary material

## Supplementary material

The supplementary material for this article can be found at http://doi.org/10.1017/S026646232510319X.
