# Supplementary material for: MAPPING current decision-making pathways and reimbursement processes for high-risk medical devices in EU/EEA member states and the UK: a scoping review
Source: Int J Technol Assess Health Care. 2025 Oct 20;41(1):e78. doi: 10.1017/S026646232510319X (PMC12592967; doi:10.1017/S026646232510319X)
Supplement: Alshaikh et al. supplementary material [file S026646232510319Xsup001.docx]

**Supplementary data**

**MAPPING CURRENT DECISION-MAKING PATHWAYS AND REIMBURSEMENT PROCESSES FOR HIGH-RISK MEDICAL DEVICES IN EU/EEA MEMBER STATES AND THE UK: A SCOPING REVIEW**

Rasha A. Alshaikh ^1,2,3,*^, Kieran A. Walsh ^1,3^, Fatma El-Komy ^1^, Susan Spillane  ^3^, Marie Carrigan  ^3^, Louise Larkin ^3^, Patricia Harrington  ^3^, Michelle O’Neill ^3^, Conor Teljeur  ^3^, Máirín Ryan^3,4,**^, Caitriona M. O’Driscoll ^1,**^.

^1^ School of Pharmacy, University College Cork, Cork, Ireland.

^2^ Faculty of Pharmacy, Tanta University, Tanta, Egypt.

^3^ Health Technology Assessment Directorate, Health Information and Quality Authority, Cork,

Ireland.

^4^ Department of Pharmacology & Therapeutics, Trinity College Dublin, Trinity Health Sciences, James Street, Dublin 8, Ireland.

^*^ Corresponding author

Rasha A. Alshaikh

School of Pharmacy,

University College Cork,

Cork,

Ireland.

Tel 00353-83-0226968

[ralshaikh@ucc.ie](mailto:ralshaikh@ucc.ie)

^**^ Denotes joint senior authorship

# Supplementary methods

## Research questions and objectives

The research questions were developed using the PCC (Population, Concept, Context) framework, guiding the identification and selection of publications relevant to these specific areas.

Supplementary Table S1. Population, Concept and Context (PCC) for the research questions

| PCC element | RQ1 | RQ2 |
| --- | --- | --- |
| Population | Stakeholders (for example, HTA bodies and ministries of health) subject to the new HTAR framework, as well as in the UK, involved in advising on and/or making reimbursement decisions for high-risk medical devices and diagnostics, including:   - National health authorities - Health Technology Assessment (HTA) agencies | |
| Context | Key decision-making pathways, stages, and oversight structures involved in the reimbursement process. | The utilization and assessment of comparative clinical safety and effectiveness data to guide the reimbursement decision. |
| Concept | - High-risk medical devices (classified as Class IIb and III under MDR Regulation 2017/745) and in vitro diagnostics (Class D under Regulation (EU) 2017/746) that are subject to joint clinical assessments under Article 7 of Regulation (EU) 2021/2282. | |

## Inclusion and exclusion criteria

The types of publications that were eligible for inclusion in this scoping review were:

- Official reports, manuals, process guidelines, methodological handbooks, methodological guidelines, policy documents, website pages, and other relevant documents from HTA agencies, national health authorities, and other policymaking bodies involved in reimbursement decisions (for example, public health insurance organizations). Each document had to provide information about the decision-making processes and oversight structures involved in reimbursement and/or the criteria used in assessing medical devices and/or in vitro diagnostics for reimbursement.
- Peer-reviewed articles or preprint publications that describe or evaluate the decision-making processes, pathways, and oversight structures for reimbursing high-risk medical devices and diagnostics, assess or analyze the effectiveness, strengths, and limitations of the current regulatory and decision-making frameworks governing the reimbursement of high-risk medical devices and in vitro diagnostics, or discuss how comparative safety and effectiveness data for high-risk medical devices and diagnostics are generated, assessed, and utilized in reimbursement decision-making. Publications from 2017 onwards, in any language, were included.
- Only documents and studies relevant to EU/EEA countries and the UK were considered, given the study’s focus on contexts affected by HTAR, MDR and IVDR.

The following exclusion criteria were employed:

- Studies and documents were excluded if they did not meet the study’s central focus on reimbursement of high-risk devices and in-vitro diagnostics within the EU/EEA and UK context. Specifically:
- Studies focused on countries outside the EU and EEA region and the UK were excluded.
- Guidelines/guidance documents focusing exclusively on methodological issues and providing no information on how these factors affect reimbursement decisions were excluded.
- Sources focused on non-high-risk devices and diagnostics that do not fall under the high-risk categories (Class IIb and III) or Class D IVD subject to joint clinical assessment were excluded. Reports that refer to medicinal products exclusively and/or all therapeutic products more generally were also excluded.
- Sources focused on disease-specific HTA processes and reimbursement conditions were excluded, except when such documents contributed broader insights into HTA practices and reimbursement decisions for high-risk medical devices.
- Sources focused on device-specific (addressing only a particular medical device or small group of devices) HTA processes and reimbursement conditions were excluded unless they provided generalizable information on HTA and reimbursement frameworks.
- Sources that exclusively focused on financial aspects of the reimbursement process without discussing HTA criteria or regulatory frameworks were excluded

## Official and organization websites search

The websites of key organizations involved in HTA and policymaking were searched between August and November 2024, as listed in Supplementary Table S2.

**Supplementary Table S2.** List of official websites that were searched for relevant reimbursement regulations or process descriptions.

| Country | | Organization | Website* |
| --- | --- | --- | --- |
| International | | EUnetHTA | <https://www.eunethta.eu/> |
|  |  | WHO | <https://www.who.int/> |
|  |  | The Pharmaceutical Pricing and Reimbursement Information (PPRI) network | <https://ppri.goeg.at/ppri_country_information> |
| 1. | **Austria** | Austrian Institute for Health Technology Assessment | <https://aihta.at/page/homepage/en> |
|  |  | Federal Ministry of Social Affairs, Health, Care and Consumer Protection | <https://www.sozialministerium.at/> |
|  |  | Umbrella Association of Social Insurance Institutions | <https://www.sozialversicherung.at/> |
| 2. | **Belgium** | Belgian Health Care Knowledge Centre | <https://kce.fgov.be/en> |
|  |  | Belgian National Institute for Health and Disability Insurance | <https://www.inami.fgov.be> |
| 3. | **Bulgaria** | Bulgarian Drug Agency | <http://www.bda.bg/> |
|  |  | National Health Insurance Fund | <http://www.nhif.bg/> |
|  |  | National council on prices and reimbursement of medicinal products | <https://ncpr.bg/en/ncprmp/about-council.html> |
| 4. | **Croatia** | Croatian Health Insurance Fund | <https://hzzo.hr/> |
|  |  | Agency for Quality and Accreditation in Health Care and Social Welfare | <https://aaz.hr/> |
|  |  | Agency for Medicinal Products and Medical Devices of Croatia | <https://www.halmed.hr/en/> |
| 5. | **Cyprus** | Ministry of Health | <https://www.moh.gov.cy/> |
|  |  | Health Insurance Organization | <https://www.gesy.org.cy/> |
| 6. | **Czech Republic** | State Institute for Drug Control | <http://www.sukl.cz/> |
|  |  | Czech Health Research Council | <http://www.azvcr.cz/> |
|  |  | Ministry of Health | <https://mzd.gov.cz/en/the-ministry-of-health/> |
| 7. | **Denmark** | Danish Medicines Agency | <https://laegemiddelstyrelsen.dk/en/> |
|  |  | Danish Health Authority | <https://www.sst.dk/en> |
|  |  | Danish Health Technology Council | <https://behandlingsraadet.dk/in-english> |
|  |  | Danish Medicines Council | <https://medicinraadet.dk/om-os/in-english> |
| 8. | **Estonia** | Estonian Health Insurance Fund | <https://www.tervisekassa.ee/en> |
|  |  | Ministry of Social Affairs | <https://www.sm.ee/en> |
|  |  | Centre for Health Technology Assessment | <https://ut.ee/en/unit-health-information-analysis> |
| 9. | **Finland** | Social Insurance Institution of Finland | <http://www.kela.fi/web/en> |
|  |  | Finnish Medicines Agency | <https://www.fimea.fi/> |
|  |  | The Ministry of Social Affairs and Health | <https://stm.fi/en/frontpage> |
|  |  | Finnish Coordinating Center for Health Technology Assessment | <https://oys.fi/fincchta/en/fincchta/> |
| 10. | **France** | National Health Insurance Fund | <https://www.ameli.fr/> |
|  |  | Haute Autorité de Santé | <https://www.has-sante.fr/> |
| 11. | **Germany** | Federal Ministry of Health | <https://www.bundesgesundheitsministerium.de/> |
|  |  | Institute for Quality and Efficiency in Health Care | <https://www.iqwig.de/en> |
|  |  | Joint Federal Committee | <https://www.g-ba.de/english/> |
| 12. | **Greece** | National Organization for Healthcare Provision | <https://www.eopyy.gov.gr/> |
|  |  | Ministry of Health | <https://www.moh.gov.gr/> |
|  |  | National Organisation of Medicines | <https://www.eof.gr/web/guest> |
| 13. | **Hungary** | National Health Insurance Fund | <http://www.neak.gov.hu/> |
|  |  | National Institute of Pharmacy and Nutrition | <https://ogyei.gov.hu/> |
| 14. | **Ireland** | Health Service Executive | <https://www.hse.ie/eng/> |
|  |  | Health Information and Quality Authority | <https://www.hiqa.ie/> |
|  |  | Health Products Regulatory Authority | <https://www.hpra.ie/> |
|  |  | National Centre for Pharmacoeconomics | <https://www.ncpe.ie/> |
| 15. | **Italy** | Italian Medicines Agency | <https://www.aifa.gov.it/> |
|  |  | Ministry of Health | <https://www.salute.gov.it/> |
|  |  | National Agency for Regional Healthcare Services | <https://www.agenas.gov.it/> |
| 16. | **Latvia** | National Health Service of Latvia | <http://www.vmnvd.gov.lv> |
|  |  | State Agency of Medicines of the Republic of Latvia | <https://www.zva.gov.lv/> |
| 17. | **Lithuania** | National Health Insurance Fund | <https://www.vlk.lt/en> |
|  |  | State Health Care Accreditation Agency | <https://vaspvt.lrv.lt/en/> |
| 18. | **Luxembourg** | National Health Fund | <https://cns.public.lu/en.html> |
|  |  | Ministry of Health, Directorate of Health/Division of Pharmacy and Medicines | <https://m3s.gouvernement.lu/en.html> |
| 19. | **Malta** | Medicines Authority | <https://medicinesauthority.gov.mt> |
|  |  | Directorate for Pharmaceutical Affairs | <https://healthservices.gov.mt/en/pharmaceutical/Pages/pharmaceutical-affairs.aspx> |
| 20. | **The Netherlands** | National Health Care Institute | <https://www.zorginstituutnederland.nl/> |
|  |  | Ministry of health, Wellbeing and Sport | <https://www.rijksoverheid.nl/ministeries/ministerie-van-volksgezondheid-welzijn-en-sport> |
| 21. | **Poland** | National Health Fund | <https://www.nfz.gov.pl> |
|  |  | Agency for Health Technology Assessment and Tariff System | <https://www.aotm.gov.pl/> |
| 22. | **Portugal** | Directorate-General for Health | <https://www.dgs.pt/> |
|  |  | National Authority for Medicines and Health Products | <https://www.infarmed.pt/> |
| 23. | **Romania** | National Health Insurance House | <http://www.cnas.ro/> |
|  |  | National Agency for Medicines and Medical Devices | <http://www.anm.ro/> |
| 24. | **Slovakia** | Ministry of Health of the Slovak Republic | <http://www.health.gov.sk/> |
|  |  | National Institute for Value and Technology in Health | <https://niho.sk/> |
| 25. | **Slovenia** | Health Insurance Institute of Slovenia | <https://www.zzzs.si/> |
|  |  | Agency for Medicinal Products and Medical Devices of the Republic of Slovenia | <https://www.jazmp.si/en> |
|  |  | Ministry of Health | <https://www.gov.si/en/state-authorities/ministries/ministry-of-health/> |
| 26. | **Spain** | Spanish Agency for Medicines and Health Products | <https://www.aemps.gob.es/> |
|  |  | Spanish Network of Agencies for Assessing National Health System Technologies and Performance, Ministry of Health | <https://redets.sanidad.gob.es/en/conocenos/quienesSomos/home.htm> |
| 27. | **Sweden** | Dental and Pharmaceutical Benefits Agency | <https://www.tlv.se/> |
|  |  | National Board of Health and Welfare | <https://www.socialstyrelsen.se/en/> |
|  |  | Swedish Agency for Health Technology Assessment and Assessment of Social Services | <https://www.sbu.se/en/> |
|  |  | National Collaboration on Medical Technology | <https://samverkanmedicinteknik.se/> |
|  |  | Swedish Medical Products Agency | <https://www.lakemedelsverket.se/sv> |
| 28. | **United Kingdom** | National Health Service | <https://www.nhs.uk/> |
|  |  | National Institute for Health and Care Excellence | <https://www.nice.org.uk/> |
|  |  | Scottish Health Technologies Group | <https://shtg.scot/> |
|  |  | Health Technology Wales | <https://healthtechnology.wales/> |
|  |  | Medicines & Healthcare products  Regulatory Agency | <https://www.gov.uk/government/organisations/medicines-and-healthcare-products-regulatory-agency> |
|  |  | Health and Social Care Board (HSCB) Northern Ireland | <https://online.hscni.net/> |
| 29. | **Liechtenstein** | Office of Public Health in Liechtenstein | <https://www.llv.li/de/landesverwaltung/amt-fuer-gesundheit> |
| 30. | **Iceland** | Icelandic Medicines Agency | <https://www.ima.is/> |
|  |  | Ministry of Health | <https://www.government.is/ministries/ministry-of-health/> |
| 31. | **Norway** | Norwegian Medical Products Agency | <https://www.dmp.no/en> |
|  |  | Norwegian Regional Health Authority | <https://www.helse-nord.no/?lang=en_US> |

* It is important to highlight that the provided URLs represent the official URL and initial starting points for the search. However, as our research progresses, we may be directed to additional organizations, websites, and reports that offer relevant information.

## Database search

The following databases were searched on September 3, 2024: Medline Complete (via EBSCOhost, the search strategy defined in supplementary Table S3), Embase (via Elsevier, the search strategy is defined in supplementary Table S4), the Cochrane Library (via Wiley, the search strategy is defined in supplementary Table S5), INAHTA Database, the search strategy is defined in supplementary Table S6).

**Supplementary Table S3. Search strategy of Medline database via EBSCOhost.**

| Database Name: Medline via EBSCOhost  Date search was run: September 02 2024 | | | | |
| --- | --- | --- | --- | --- |
| # | **Query** | **Limiters/Expanders** | **Last Run Via** | **Results** |
| S21 | S5 AND S10 AND S19 | Limiters - Publication Date: 20170101-20241231 Expanders - Apply equivalent subjects Search modes - Proximity | Interface - EBSCOhost Research Databases Search Screen - Advanced Search Database - MEDLINE Complete | 297 |
| S20 | S5 AND S10 AND S19 | Expanders - Apply equivalent subjects Search modes - Proximity | Interface - EBSCOhost Research Databases Search Screen - Advanced Search Database - MEDLINE Complete | 1,225 |
| Concept 3: Decision making pathway | | | | |
| S19 | S11 OR S12 OR S13 OR S14 OR S15 OR S16 OR S17 OR S18 | Expanders - Apply equivalent subjects Search modes - Proximity | Interface - EBSCOhost Research Databases Search Screen - Advanced Search Database - MEDLINE Complete | 74,308 |
| S18 | AB ( ("decision making" OR "decision-making") n2 (process* OR pathway* OR framework*) ) OR TI ( ("decision making" OR "decision-making") n2 (process* OR pathway* OR framework*) ) | Expanders - Apply equivalent subjects Search modes - Proximity | Interface - EBSCOhost Research Databases Search Screen - Advanced Search Database - MEDLINE Complete | 31,391 |
| S17 | AB ( "oversight structure*" OR "governance structure*" ) OR TI ( "oversight structure*" OR "governance structure*" ) | Expanders - Apply equivalent subjects Search modes - Proximity | Interface - EBSCOhost Research Databases Search Screen - Advanced Search Database - MEDLINE Complete | 1,283 |
| S16 | AB joint n1 assessment* OR TI joint n1 assessment* | Expanders - Apply equivalent subjects Search modes - Proximity | Interface - EBSCOhost Research Databases Search Screen - Advanced Search Database - MEDLINE Complete | 1,414 |
| S15 | AB ( "health technology assessment*" OR HTA OR HTAR ) OR TI ( "health technology assessment*" OR HTA OR HTAR ) | Expanders - Apply equivalent subjects Search modes - Proximity | Interface - EBSCOhost Research Databases Search Screen - Advanced Search Database - MEDLINE Complete | 9,098 |
| S14 | AB ( "medical device* regulation*" OR (MDR n2 regulation*) OR "EU MDR" OR (regulation* n2 "2017/745") OR (regulation* n2 "2021/2282") ) OR TI ( "medical device* regulation*" OR (MDR n2 regulation*) OR "EU MDR" OR (regulation* n2 "2017/745") OR (regulation* n2 "2021/2282") ) | Expanders - Apply equivalent subjects Search modes - Proximity | Interface - EBSCOhost Research Databases Search Screen - Advanced Search Database - MEDLINE Complete | 487 |
| S13 | (MH. "Health Care Rationing") | Expanders - Apply equivalent subjects Search modes - Proximity | Interface - EBSCOhost Research Databases Search Screen - Advanced Search Database - MEDLINE Complete | 12,144 |
| S12 | (MH "Decision Making, Organizational") | Expanders - Apply equivalent subjects Search modes - Proximity | Interface - EBSCOhost Research Databases Search Screen - Advanced Search Database - MEDLINE Complete | 11,247 |
| S11 | (MH "Technology Assessment, Biomedical+") | Expanders - Apply equivalent subjects Search modes - Proximity | Interface - EBSCOhost Research Databases Search Screen - Advanced Search Database - MEDLINE Complete | 12,418 |
| Concept 2: reimbursement | | | | |
| S10 | S6 OR S7 OR S8 OR S9 | Expanders - Apply equivalent subjects Search modes - Proximity | Interface - EBSCOhost Research Databases Search Screen - Advanced Search Database - MEDLINE Complete | 884,126 |
| S9 | AB ( reimburs* OR fund* ) OR TI ( reimburs* OR fund* ) | Expanders - Apply equivalent subjects Search modes - Proximity | Interface - EBSCOhost Research Databases Search Screen - Advanced Search Database - MEDLINE Complete | 608,604 |
| S8 | (MH. "Health Expenditures+") | Expanders - Apply equivalent subjects Search modes - Proximity | Interface - EBSCOhost Research Databases Search Screen - Advanced Search Database - MEDLINE Complete | 26,895 |
| S7 | (MH "Reimbursement Mechanisms+") | Expanders - Apply equivalent subjects Search modes - Proximity | Interface - EBSCOhost Research Databases Search Screen - Advanced Search Database - MEDLINE Complete | 38,672 |
| S6 | (MH "Costs and Cost Analysis+") | Expanders - Apply equivalent subjects Search modes - Proximity | Interface - EBSCOhost Research Databases Search Screen - Advanced Search Database - MEDLINE Complete | 272,324 |
| Concept 1: High risk medical devices | | | | |
| S5 | S1 OR S2 OR S3 OR S4 | Expanders - Apply equivalent subjects Search modes - Proximity | Interface - EBSCOhost Research Databases Search Screen - Advanced Search Database - MEDLINE Complete | 2,144,850 |
| S4 | TX ("High* risk" OR high-risk OR "Class III" OR "class IIb") AND ("medical device*" OR diagnostics) | Expanders - Apply equivalent subjects Search modes - Proximity | Interface - EBSCOhost Research Databases Search Screen - Advanced Search Database - MEDLINE Complete | 168,853 |
| S3 | MH "Diagnostic Techniques and Procedures+/AE/IS/ST") | Expanders - Apply equivalent subjects Search modes - Proximity | Interface - EBSCOhost Research Databases Search Screen - Advanced Search Database - MEDLINE Complete | 382,767 |
| S2 | (MH "Equipment Safety") | Expanders - Apply equivalent subjects Search modes - Proximity | Interface - EBSCOhost Research Databases Search Screen - Advanced Search Database - MEDLINE Complete | 10,458 |
| S1 | (MH "Equipment and Supplies+") | Expanders - Apply equivalent subjects Search modes - Proximity | Interface - EBSCOhost Research Databases Search Screen - Advanced Search Database - MEDLINE Complete | 1,692,217 |

**Supplementary Table S4. Search strategy of Embase database via Elsevier.**

| Database Name: Embase via Elsevier  Date search was run: September 03 2024 | | |
| --- | --- | --- |
| # | **Query** | **Results** |
| #22 | #5 AND #10 AND #20 AND [2017-2024]/py | 725 |
| #21 | #5 AND #10 AND #20 | 1,873 |
| #20 | #11 OR #12 OR 13 OR #14 OR #15 OR #16 OR #17 OR #18 OR #19 | 67,802 |
| #19 | (('decision making' OR 'decision-making') NEAR/2 (process* OR pathway* OR framework*)):ab,ti | 37,489 |
| #18 | 'oversight structure*':ab,ti OR 'governance structure*':ab,ti | 1,538 |
| #17 | (joint NEAR/1 assessment*):ab,ti | 953 |
| #16 | 'health technology assessment*':ab,ti OR hta:ab,ti OR htar:ab,ti | 15,387 |
| #15 | (regulation* NEAR/2 ('2017/745' OR '2021/2282')):ab,ti | 96 |
| #14 | (mdr NEAR/2 regulation*):ab,ti | 241 |
| #13 | 'medical device* regulation*':ab,ti OR 'eu mdr':ab,ti | 621 |
| #12 | 'organizational decision making'/exp | 111 |
| #11 | 'biomedical technology assessment'/exp | 18,482 |
| #10 | #8 OR #9 OR #10 OR #11 | 1,115,423 |
| #9 | reimburs*:ab,ti OR fund*:ab,ti | 692,183 |
| #8 | 'health care cost'/exp | 358,366 |
| #7 | 'reimbursement'/exp | 69,033 |
| #6 | 'cost'/exp | 427,766 |
| #5 | #1 OR #2 OR #3 OR #4 | 5,960,265 |
| #4 | ('high* risk':ti,ab,kw OR 'high risk':ti,ab,kw OR 'class iii':ti,ab,kw OR 'class iib':ti,ab,kw) AND ('medical device*':ti,ab,kw OR diagnostics:ti,ab,kw) | 5,506 |
| #3 | 'diagnostic procedure'/mj | 18,193 |
| #2 | 'device safety'/mj | 3,001 |
| #1 | 'devices'/exp | 5,937,142 |

**Supplementary Table S5. Search strategy of the Cochrane Library.**

| Database Name: The Cochrane Library  Date search was run: September 03 2024 | | |
| --- | --- | --- |
| # | **Query** | **Results** |
| #1 | (("High risk" OR high-risk OR "Class III" OR "class IIb") AND ("medical device" OR "medical devices" OR diagnostics)) (Word variations have been searched) | 8150 |
| #2 | (reimburs* OR fund*):ti,ab,kw (Word variations have been searched) | 45822 |
| #3 | (medical NEXT device* NEXT regulation*):ti,ab,kw (Word variations have been searched) | 15 |
| #4 | (MDR NEAR/2 regulation*):ti,ab,kw (Word variations have been searched) | 4 |
| #5 | ("EU MDR"):ti,ab,kw (Word variations have been searched) | 4 |
| #6 | (regulation* NEAR/2 ("2017/745" OR "2021/2282")):ti,ab,kw (Word variations have been searched) | 4 |
| #7 | ("health technology assessment" OR "health technology assessments" OR HTA OR HTAR):ti,ab,kw (Word variations have been searched) | 1552 |
| #8 | (joint NEAR/1 assessment*):ti,ab,kw (Word variations have been searched) | 131 |
| #9 | ("oversight structure" OR "governance structure" OR "oversight structures" OR "governance structures"):ti,ab,kw (Word variations have been searched) | 23 |
| #10 | (("decision making" OR "decision-making") NEAR/2 (process* OR pathway* OR framework*)):ti,ab,kw (Word variations have been searched) | 1459 |
| #11 | #3 OR #4 OR #5 OR #6 OR #7 OR #8 OR #9 OR #10 | 3184 |
| #12 | #1 AND #2 AND #11 with Cochrane Library publication date from Jan 2017 to present | 16 |

**Supplementary Table S6. Search strategy of the INAHTA Database.**

| Database Name: INAHTA Database  Date search was run: September 03 2024 | |
| --- | --- |
| Search Strategies | Search 1: (medical devices)[abs] AND (high risk OR high-risk)[abs] AND (reimburs*)  Search 2: ((medical devices)[abs] AND (high risk OR high-risk)[abs] )[abs] AND (fund*)[abs]  Search 3: "high risk medical devices" |

**Supplementary Table S7. Results and search date of database search.**

| **Databases** | **Number of results** | **Date searched** |
| --- | --- | --- |
| Medline Complete | 297 | 03/09/2024 |
| Embase | 725 | 03/09/2024 |
| The Cochrane Library | 16 | 03/09/2024 |
| INAHTA Database | 7 | 03/09/2024 |
| Total | 1045 | |
| Total after duplicates removed | **953** | |

**Supplementary Table S8.** Templates for data extraction from the included sources.

| **Country *** | | |
| --- | --- | --- |
| *Document name/title* | **Source A** | **Source B** |
| *Document identifier (for example, PMID or DOI for published articles)* |  |  |
| *URL* |  |  |
| *Organization* |  |  |
| *Authors* |  |  |
| *Funding* |  |  |
| *Date of publication/ Date of access* |  |  |
| *Original language of the document* |  |  |
| *Translated (Yes/No)* |  |  |
| *Summary of the methods (for publications)* |  |  |
| ***RQ1 What are the key decision-making pathways, stages of decision-making and oversight structures involved in reimbursing high-risk medical devices and diagnostics in the EU/EEA and the UK.*** | | |
| 1.1. What are the different schemes or pathways of reimbursement of high-risk medical devices and in-vitro diagnostics. |  |  |
| 1.2. Who initiates the process of reimbursement in each pathway |  |  |
| 1.3. What are the stages of decision-making and oversight structures involved in each stage for each pathway. |  |  |
| 1.4. What are the factors taken into consideration during reimbursement decision |  |  |
| 1.5. Are there specific provisions for emergency use or expedited pathways for particular devices? If yes, what are they. What are the different schemes or pathways of reimbursement |  |  |
| 1.6. Are there any regional variations in the reimbursement process within the country. |  |  |
| ***(RQ2) How are the comparative safety and effectiveness data of high-risk medical devices and diagnostics used for reimbursement decisions in EU/EEA and the UK.*** | | |
| 2.1. How is comparative safety and effectiveness evidence data being considered for reimbursement decisions |  |  |
| 2.2. How is the uncertainty of comparative safety and effectiveness addressed during decision decision-making (for example, is there a provisional approval, approval for research only, approval with managed access or the capacity to require and/or fund clinical trials). |  |  |
| 2.3. After reimbursement, is there a monitoring period? If yes, describe the different stages and oversight structures involved in the process until a final reimbursement decision is made. |  |  |
| 2.4. How is the uncertainty of comparative safety and effectiveness addressed during decision-making. |  |  |

## Data validation

The data validation process was conducted between December 2024 and January 2025. Representatives from HTA organizations, ministries of health, or other relevant reimbursement decision-making bodies were directly contacted (via email) and asked to review and provide feedback on the national information. A list of organizations that provided data validation is presented in Supplementary Table S9.

**Table S9. List of countries and official organizations from which information verification was received.**

| **Country** | | | **National organizations that provided information verification** |
| --- | --- | --- | --- |
| 1 | Austria | | Austrian Institute for Health Technology Assessment (AIHTA) |
| 2 | Belgium | | National Institute for Health and Disability Insurance (RIZIV-INAMI) |
| 3 | Bulgaria | | National council on prices and reimbursement of medicinal products |
| 4 | Croatia | | Croatian Agency for Medicinal Products and Medical Devices of Croatia |
| 5 | Cyprus | | Medical and Public Health Services at the Ministry of Health |
| 6 | Czech Republic | | State Institute for Drug Control (SÚKL) |
| 7 | Denmark | | Danish Health Technology Council (DHTC) |
| 8 | Estonia | | Estonian Ministry of Social Affairs |
| 9 | Finland | | Finnish Medicines Agency (Fimea)  Finnish Coordinating Center for Health Technology Assessment (FinCCHTA) |
| 10 | France | | French National Authority for Health (Haute Autorité de Santé; HAS) |
| 11 | Germany | | Institute for Quality and Efficiency in Healthcare (Institut für Qualität und Wirtschaftlichkeit im Gesundheitswesen; IQWiG) |
| 12 | Greece | | National Organization For Health Care Services (EOPYY) |
| 13 | Hungary | | National Centre for Public Health and Pharmacy (Nemzeti Népegészségügyi és Gyógyszerészeti Központ; NNGYK) |
| 14 | Ireland | | Health Information and Quality Authority (HIQA) |
| 15 | Italy | | National Agency for Regional Health Services (Agenas) |
| 16 | Latvia | | State Agency of Medicines of the Republic of Latvia |
| 17 | Lithuania | | State Accreditation Service for Health Care Activities under the Ministry of Health of the Republic of Lithuania (VASPVT).  National Health Insurance Fund (VLK) |
| 18 | Malta | | Directorate for Pharmaceutical Affairs (DPA) |
| 19 | The Netherlands | | National Health Care Institute (ZIN) |
| 20 | Portugal | | National Authority of Medicines and Health Products, I.P (INFARMED) |
| 21 | Romania | | National Agency For Medicines And Medical Devices Of Romania |
| 22 | Slovakia | | National Institute for Value and Technologies in Healthcare (NIHO) |
| 23 | Slovenia | | Agency For Medicinal Products And Medical Devices Of The Republic Of Slovenia (JAZMP) |
| 24 | Spain | | The Spanish Agency of Medicines and Medical Devices (AEMPS)  Spanish Network of Agencies for the Evaluation of Health Technologies and Services of the National Health System (RedETS) |
| 25 | Sweden | | The Dental and Pharmaceutical Benefits Agency (Tandvårds- och läkemedelsförmånsverket; TLV) |
| 26 | United Kingdom | England | National Institute for Health and Care Excellence (NICE) |
|  |  | Scotland | Scottish Health Technologies Group (SHTG) |
|  |  | Wales | Health Technology Wales (HTW) |
|  |  | Northern Ireland | Department of Health |
| 27 | Norway | | Norwegian Medical Products Agency (NOMA) |

# Supplementary results

## Key oversight structures involved in decision-making on patient access to medical devices in the EU/EEA/UK

Different stakeholders in each country can initiate the reimbursement process for MD and IVD. In at least 17 countries (out of 23), the HTDs or their legal representatives can directly initiate the assessment and reimbursement process for certain types of MD/IVD. Other stakeholders (such as hospitals, patient associations, horizon scanning programmes, HTA bodies or the Ministry of Health members, and scientific and professional associations) can also initiate reimbursement requests in at least 14 countries (Supplementary Table S10).

After proposal submission, a formal prioritization process before assessment was reported in at least eight countries (Supplementary Table S10). In six of these eight countries, prioritization is conducted by an independent body or committee operating separately from the HTA-performing organization (for example, the Estonian HTA Expert Council and MTP Council in Sweden; Supplementary Table S10). The most common prioritization criteria include clinical and patient outcomes (expected patient benefit, disease severity, patient number, and comparative effectiveness of the technology), economic and resource impact (financial implications and resource use), technological innovation and the presence of alternatives, scientific evidence uncertainty and associated risks.

Supplementary Table S10. Overview of key oversight structures involved in the initiation and prioritization of reimbursement of high-risk MD/IVD across the EU/EEA/UK as of November 2024.

| **Country (reimbursement pathway)** | | **Initiation of the reimbursement process and prioritization** | |
| --- | --- | --- | --- |
|  |  | **Initiation** | **Prioritization** |
| Austria (Applications for new reimbursement groups under LKF). | | Proposals can be submitted by public hospitals and regional hospital federations to the **Austrian Ministry of Health.** | Assessment proposals are prioritized by the **Austrian Ministry of Health** according to the (a) potential for true innovation, (2) financial implications, (3) the number of patients, and (4) the level of uncertainty or potential risks. |
| Belgium | | For MD, manufacturers or distributors submit the request to the **CTIIMH-CRIDMI** in **INAMI-RIZIV**. Minister of Social Affairs, professional associations and members of the CTIIMH-CRIDMI can also initiate the request. | No information^†^ |
|  |  | For IVD, manufacturers or distributors submit the request to the **CTM.** | The admissibility of the request is evaluated, but without a prioritization process. |
| Croatia | | The manufacturer, representative, or distributor submits the request to **CHIF’s** expert committees and initiates the assessment.  MoH or hospital management can also initiate the process. | Unclear |
| Czech Republic (Separately Billed Materials “ZUM”) | | Unclear | Unclear |
| Denmark | | The manufacturer can submit the evaluation request.^*^  Regional governments and hospital management can also submit the request. | Unclear |
| Estonia | | Manufacturers or representatives make submissions for outpatient device reimbursement to the **EHIF**.  Professional Association of Healthcare Providers can only make proposals regarding reimbursement criteria.  The submission process for inpatient high-risk devices is unclear. | All submitted requests will be assessed. |
| Finland | | Unclear | Unclear |
| France | | Health technology developers submit application dossiers to the **Minister of Health** and send them simultaneously to **CNEDiMTS** for HTA evaluation. | Unclear |
| Germany  (for high-risk devices with a novel mechanism of action) | | The hospital submits an application to **InEK** for reimbursement.  In parallel, the hospital, in agreement with the medical device manufacturer, submits the dossier to the **G-BA** for assessment. | Unclear |
| Hungary | | The manufacturer submits the application for reimbursement to the National Health Insurance Fund **(NEAK).**  The ability of other stakeholders to initiate the process is unclear. | Unclear |
| Ireland | | The **Department of Health** or the **HSE** may initiate the process by requesting an HTA. | Unclear |
| Italy | | The process is initiated when a new MD is integrated into the system by collaboration among the scientific community, physicians, patient associations, and manufacturers.  The ability of specific stakeholders to initiate the process is unclear. | Unclear |
| Lithuania | | Only public or private legal entities seeking reimbursement can submit requests to **VASPVT**.  Then, **VASPVT** will submit information on the applications received to the **HTA Committee** of the **Ministry of Health**. | The **HTA Committee** prioritizes the evaluation according to a score-based system that considers (a) disease severity, (b) scientific validity, (c) treatment alternatives and (d) the expected impact.  Technologies that score 21-30 points (1^st^ priority) or 11-20 points (2^nd^ priority) are submitted for HTA. Technologies that score 0-10 points (3^rd^ priority) are not assessed. |
| Netherlands | | The manufacturer, health insurers, scientific associations, patient organizations, or **ZIN** itself can initiate the assessments only in case of uncertainty about whether or not a treatment can be reimbursed from the Health Care Package. | Unclear |
| Poland | | The manufacturer submits an application for reimbursement to **MoH**.  The ability of other stakeholders to initiate the process is unclear. | Unclear |
| Portugal | | The manufacturer or its legal representative submits a financing request to **INFARMED** to initiate the process. | The financing request is validated in relation to a set of legal requirements (for example, specific technical characteristics of the medical device). |
| Slovakia | | The registration holder (for example manufacturer) applies for medical device categorization to the **Ministry of Health (MZSR).**  The ability of other stakeholders to initiate the process is unclear. | Unclear |
| Spain  (updating the content of the Common Portfolio of Services) | | Proposals are submitted by the Ministry of Health or the regional health administrations on their own initiative or at the request of interested third parties (for example, insurance companies and manufacturers). | Technologies are prioritized by the **Committee for Provision, Insurance and Financing**, using a scoring tool, the PriTec tool (<https://pritectools.sergas.gal/>).  The prioritization criteria are clustered in five domains: (a) the disease (b) comparative results, (c) comparative costs (d) repercussions of implementation implications, and (e) dissemination issues. |
| Sweden  (joint cooperation model for national assessment and procurement of medical technologies) | | The process is initiated through horizon scanning programs (that involve consultation with experts) by the **MTP Council** to identify products that could be relevant for the national-level introduction. | The **MTP Council** prioritizes the technologies for assessment. Criteria include (a) severity of conditions, (b) expected patient benefit, and (c) resource impact. |
| UK | England (new health technologies) | Topics are notified by the sponsors of medical technologies or other stakeholders (for example, NIHR, NHS, health, royal colleges, patients and healthcare organizations). | **NICE’s prioritization board** evaluates notified technologies and routes them to suitable assessment programs.  Prioritization criteria include cost effects and the potential disruptiveness of the expected benefit. |
|  |  |  |  |
|  | Scotland | Any member of the public can initiate the process by submitting a topic for HTA. | Unclear |
|  | Wales | Any member of the public can initiate the process by proposing a topic for HTA. | **HTW** assesses the proposed topics against appraisal criteria and produces a Topic Exploration Report. |
| Norway | | **Manufacturers** can submit proposals for the assessment.  Topics can also be identified through **NOMA’s** early awareness system and through proposals by **hospitals**, **patient associations**, or **the public**. | Priority assessment is conducted by the  **commissioner of HTA** (**“Ordering Forum”**), which consists of the chief medical directors from each of the four RHAs^***^.  The prioritization is based on (a) the impact on healthcare system, (b) potential benefits, (c) associated risks, and (d) budgetary effect.  The **commissioner** decides if the MD should be evaluated through a national HTA or mini-HTA or not assessed. |

**LKF:** The Austrian procedure and diagnosis-oriented groups. **STA:** Single Technology Assessment. **MTA:** Multiple Technology Assessment. **CTIIMH-CRIDMI**: The Commission of Reimbursement of Implants and Invasive Medical Devices. **INAMI-RIZIV:** National Institute for Health and Disability Insurance. **KCE**: Belgian Health Care Knowledge Centre. **CTM:** Medical Technical Council. **CHIF:** Croatian Health Insurance Fund. **MoH:** Ministry of Health. **EHIF**: Estonian Health Insurance Fund. **Kela**: Social Insurance Institution of Finland. **CNEDiMTS:** Medical Device and Health Technology Evaluation Committee. **G-BA**: Federal Joint Committee in Germany. **InEK:** Hospital Remuneration System. **SHI:** Statutory Health Insurance in Germany. **IQWiG**: The Institute for Quality and Efficiency in Healthcare. **FHC:** Federal Healthcare Commission. **NEAK:** National Health Insurance Fund in Hungary. **NNGYK:** National Centre for Public Health and Pharmacy in Hungary.**TéF:** Technology Assessment Department in Hungary. **HIQA:** Health Information and Quality Authority. **AGENAS:** National Agency for Regional Healthcare Services. **VASPVT**: State Accreditation Service for Health Care Activities under the Ministry of Health of the Republic of Lithuania. **VLK:** National Health Insurance Fund in Lithuania. **ZIN:** the National Health Care Institute in the Netherlands. **AOTMiT:** Agency for Health Technology Assessment and Tarification. **INFARMED:** the National Authority for Medicament and Health Products. **NIHO:** National Institute For Value And Technologies In Healthcare. **MZSR:** The Ministry of Health of the Slovak Republic. **RedETS:** Spanish Network of Health Technology Assessment Agencies. **PriTec:** Prioritisation Technology Scoring Tool. **MTP Council**: Medical Technology Product Council in Sweden. **NOMA:** Norwegian Medical Products Agency (previously called the Norwegian Medicines Agency). **NICE:** National Institute for Health and Care Excellence (England). **MTEP:** Medical Technologies Evaluation Programme (England). **DAP:** Diagnostics Assessment Programme (England). **HTW:** Health Technology Wales. **SHTG**: Scottish Health Technologies Group. **NHS**: National Health Service (UK).

^*^ Before 2023, manufacturers could only submit assessment proposals if they demonstrated that the use of the technology would be cost-neutral or cost-saving. However, on February 13, 2023, this requirement was removed.

^†^ “Unclear" was used when some information was available but lacked sufficient detail to describe how prioritisation occurs. "No information" was used when no relevant description of prioritisation processes could be identified.

## The role of HTA in reimbursement pathways of high-risk MD and IVD

For the countries with any form of HTA for high-risk MD/IVD in place (23 countries), the level to which HTA is integrated into reimbursement frameworks and criteria for HTA assessment varies significantly. Extended information for each of these countries is provided in Supplementary Table S11, which provides an overview of key national reimbursement mechanisms for high-risk MD/IVD, the role of HTA in each country’s reimbursement process, and factors considered in HTA recommendations and final decisions.

For 11 countries, HTA is a formal element in specific national reimbursement frameworks for certain categories of MD/IVD. The assessments can be based on the information provided by the manufacturer, evidence synthesis from the HTA-performing body, or both. For instance, Austria, Germany, and France rely heavily on adapted national Diagnosis-Related Group (DRG) systems to reimburse high-risk medical devices in inpatient settings. DRG systems are specific hospital payment models that group patient cases with similar clinical characteristics and expected resource use, assigning a fixed reimbursement amount per case. These three countries also have an additional system for reimbursing medical devices (or procedures/interventions, including the device) that are not included in the DRG or must be reimbursed under their brand name. The inclusion of a new DRG group, the introduction of high-risk medical devices with a novel mechanism of action, or the inclusion of specific devices under a brand name might necessitate a mandatory form of HTA for reimbursement decisions, with unique criteria being assessed in each case (Supplementary Table S11).

In contrast, eight countries have adopted an optional/conditional role for HTA of medical devices, where HTA is not a formal component of any reimbursement pathway, and the devices might still be reimbursed without HTA advice. However, HTA might still be carried out for specific devices or under special circumstances on an ad hoc basis upon request from decision-makers. For instance, in Estonia and Croatia, high-risk devices are reimbursed under the Health Insurance Fund list regulated by national legislation. HTA is optional and is not a formal step of the reimbursement pathway. In some cases, HTA can be requested from the University of Tartu HTA Centre in Estonia,(1,2) or undertaken by the Ministry of Health in Croatia.(3–5) The generated HTA recommendations are not legally binding and only aim to support policymakers at a national level. For four countries, it was unclear if the HTA is a formal part of any reimbursement pathways for MD/IVD.

Supplementary Table S11. Overview of key national reimbursement mechanisms of MD/IVD that involve HTA, the role of HTA in the process and utilization of safety and effectiveness evidence for recommendation and reimbursement decisions for selected countries across the EU/EEA/UK as of November 2024.

| **Country** | | **Main reimbursement mechanisms for MD and IVD** | **Role of HTA in the process** | **Criteria considered for HTA and/or reimbursement recommendations** | **Ref.** |
| --- | --- | --- | --- | --- | --- |
| **Austria** | | **1. Procedure and diagnosis-oriented groups (Leistungsorientierte Krankenanstaltenfinanzierung; LKF)** for MD in the inpatient sector.  **1.1. Individual medical services catalogue (Medizinische Einzelleistungen; MEL)** for specific services that receive an add-on tariff. | Selected interventions must undergo HTA by **AIHTA** to introduce new reimbursement groups within the system.  FHC considers the HTA results when making reimbursement decisions. | The HTA recommendations consider:   - Comparative effectiveness (added benefit). - Quality appraisals of effectiveness and safety data.   The reimbursement decisions consider:   - HTA results. - Quality assurance and organizational aspects - Procedural aspects | (6,7) |
| **Belgium** | | **1. Main list of reimbursable medical devices (the List).** | Invasive medical devices, including implants, must be evaluated by the CTIIMH-CRIDMI for reimbursement (except MD for oral cavity or facial area with an intraoral or extraoral visible part). | The HTA and reimbursement decisions consider some or all of these five criteria:   - Therapeutic value - Price and reimbursement level. - Importance in medical practice. - Impact on healthcare costs. - Cost-effectiveness. | (8–14) |
|  |  | **2. Nominative list** for selected brand names of reimbursable devices. |  |  |  |
|  |  | **3. Reimbursement of IVD not linked to a medicinal product.** | The application for reimbursement must undergo an appraisal by evaluators at CTM. | The reimbursement depends on the available budget and the appraisal results. |  |
| **Croatia** | | **Basic and additional lists** for reimbursement of medical devices covered by compulsory health insurance. | HTA is not a formal part of the reimbursement of medical devices. However, CHIF appraises the application.  HTA (by MoH) may be performed ad-hoc, and the recommendation is not legally binding when done. | The reimbursement decisions are based on the manufacturer submission, which should include:   - Expert opinion on the comparative effectiveness of the device. - Proof of the technical characteristics and the demonstration of different and/or more efficient mechanisms of action. - Budget impact analysis in certain cases. | (3,4,15) |
| **Czech Republic** | | **Reimbursement for separately billed materials (ZUM).** | It’s unclear if HTA is a formal part of any reimbursement pathway. | In the case of reimbursement of an innovative medical device, a Budget Impact Analysis from the payer's perspective is required. | (16,17) |
| **Denmark** | | **1. General reimbursement of medical devices** | It’s unclear if HTA is a formal part of any reimbursement pathway. The DHTC performs HTA upon application of the manufacturer or health authorities' request.  The HTA recommendations are not legally binding for the regional governments, but they are expected to follow them. | HTA recommendations consider:   - The device is better than the comparator(s) (in terms of clinical effectiveness) and is cost-reducing, cost-neutral or considered cost-effective). - Cost-effectiveness (the device is considered clinically equivalent to the comparator(s) at a minimum and is cost-reducing or cost-neutral. | (18,19) |
| **Estonia** | | **1.** **Positive health service list** for reimbursement of high-risk MD in the inpatient settings. | HTA is not a formal part of the reimbursement of medical devices. However, the EHIF assesses requests to amend the lists.  HTA (by the University of Tartu HTA Centre) may be performed ad-hoc, and the recommendations may be used to inform decision-making. | The reimbursement decisions consider:   - Cost-effectiveness. - Medical necessity in Estonia. | (1,2) |
|  |  | **2. Positive list of medical devices** for reimbursement of high-risk MD for outpatient settings. |  |  |  |
| **Finland** | | No information on different national mechanisms of reimbursement of MD/IVD. | It’s unclear if HTA is a formal part of any reimbursement pathway.  The legal mandate of HTA recommendations is also unclear. | HTA recommendations by FinCCHTA may consider:   - Clinical effectiveness. - Costs and financial impact. - Safety. - Ethical considerations. - Organizational aspects, social effects and legislative implications. | (20–22) |
| **France** | | **1.** **DRGs** for reimbursement of most MD for individual use in the inpatient settings.  **1.1.** **Positive intra-DRG lists** for reimbursement of specific MD under a brand name within the DRG. | HTA (by CNEDiMTS within HAS) is a formal part for nine specific categories of MD before they can be reimbursed under the “positive intra-DRG list”.^*^  HTA recommendations are the basis of reimbursement decisions. | HTA recommendations consider safety and effectiveness evidence for devices in the specified categories^*^, as they must meet at least one of the following requirements:   - Validation of clinical effectiveness. - Definition of particular technical specifications. - Assessment of the comparative effectiveness. | (23,24) |
|  |  | **2.** **LPPR (Liste des Produits et Prestations Remboursables)** for medical devices and services eligible for reimbursement in private settings, either at home or in private practices.  **2.1. The additional list within the LPPR** includes the devices used in healthcare organizations that qualify for reimbursement outside the standard DRGs. | Medical devices can be included in the LPPR list under a generic description (default inclusion) or brand name. The inclusion under a generic description is defined on the LPPR after the CNEDiMTS has performed a multiple technology assssment. After that, any medical devices corresponding to a generic description do not require further HTA for reimbursement. For inclusion under a brand name, a single technology assessment must be performed.  HTA recommendations are the basis of reimbursement decisions. | HTA recommendations by CNEDiMTS must deliver an opinion using two main components:   - The actual clinical benefit (ACB). - The clinical added value (CAV).   If the ACB is sufficient, the CNEDiMTS must then also assess the CAV compared to the most clinically relevant comparator.  If the device is expected to have a significant economic impact and if the manufacturer claims a CAV of I to III, a health economic assessment is required. | (23–27) |
| **Germany** | | **1. The German DRG system** for reimbursement of devices in the inpatient setting. | InEK must conduct assessments for reimbursement of MD that involves a new mode of action or is not represented by an existing DRG category.  HTA recommendations are the basis of reimbursement decisions. | The reimbursement decisions consider the following aspects:   - The device’s costs. - The device’s clinical value. | (28–30) |
|  |  | **2. Reimbursement of any new diagnostic or treatment procedure with a novel mechanism of action and involving high-risk (class IIb & III) or active implantable MD**.^**^ | HTA (benefit assessment; §137h SGB V) must be performed for these devices.  HTA recommendations are the basis of reimbursement decisions. | The HTA recommendations only consider the following aspect:   - Added benefit (considering only patient-relevant outcomes, including mortality, morbidity, quality of life, and side effects).   Cost-effectiveness is not formally assessed for medical devices or procedures in Germany | (31–34) |
|  |  | **3. SHI reimbursement for MD in the outpatient sector.** | HTA must be performed for new interventions (including the device) before reimbursement decision-making. | The HTA recommendations and reimbursement decisions consider whether the new intervention (including the device) demonstrates sufficient comparative effectiveness. | (31) |
| **Hungary** | | **General reimbursement for medical devices by the National Health Insurance Fund.** | HTA, in the form of technology appraisal of the manufacturer submission, must be performed (by TéF) to make reimbursement decisions on any new medical device. | HTA recommendations consider the following aspects:   - Health care priorities.   - The severity of the disease.   - Equality.   - Cost-effectiveness and quality of Life.   - Aggregated budget impact   - National and international professional opinion. | (35) |
| **Ireland** | | **Reimbursement of medical devices by HSE.** | HTA is not a formal part of any reimbursement pathway for high-risk MD/IVD. However, it may be conducted for certain medical devices and diagnostics at the request of the Department of Health or the HSE usually as a component of an innovative national programme | HTA advice considers the following domains:   - Description of technology - Burden of disease - Clinical effectiveness and safety - Cost effectiveness and budget impact - Organizational aspects - Patient and social aspects - Ethical and medicolegal aspects | (36) |
| **Italy** | | **DRG system** for reimbursement of devices in the inpatient setting. | HTA is not a formal part of any reimbursement pathways.  No HTA is needed for reimbursement under standard DRG codes. Establishing a new DRG code typically involves collaboration among the scientific community, physicians, patient associations, and manufacturers. | Unclear | (29,37,38) |
| **Lithuania** | | **General reimbursement for medical devices by the national health insurance fund**. | VASPVT and the HTA committee assess certain devices for inclusion into the reimbursement list. | HTA conducted by the VASPVT assesses the following:   - Technology purpose and use in other countries. - Health problem. - Clinical effectiveness. - Safety. - Economic aspects and benefits compared to comparators. - Social, legal, and ethical aspects. | (39–41) |
| **Netherlands**^†^ | | **Reimbursement for medical devices by the national health insurance fund.** | HTA is not a formal part of reimbursement pathways, as MD/IVD can be reimbursed without HTA assessment by ZIN. However, ZIN provides HTA in cases of disagreement over MD/IVD reimbursement between physicians, patients, and insurers regarding the treatment eligibility or effectiveness. Health insurers can only reimburse such treatments under basic insurance if ZIN provides a positive recommendation. | HTA and reimbursement recommendations consider the following aspects:   - The evidence of added value (for example, improved survival and quality of life) - Alignment with legal criteria in daily practice. - In certain cases, cost-effectiveness and broader considerations like patient and societal benefits might be considered. | (42–44) |
| **Poland** | | **General reimbursement for medical devices by the national health insurance.** | It’s unclear if HTA is a formal part of any reimbursement pathway. For certain devices, AOTMiT may perform HTA. However, the AOTMiT recommendation is not obligatory to be implemented throughout Poland. | HTA recommendations consider the following aspects:   - Health problem. - Target population. - Comparative effectiveness. - Safety assessments. - Non-clinical effects. - Economic impact. | (45–49) |
| **Portugal** | | **General reimbursement for medical devices by the National Health Services.** | For MD/IVD, the reimbursement request must be validated in relation to the device's technical requirements. Further opinion (HTA) may be requested from CATS at Infarmed to make reimbursement decisions. | HTA recommendations consider the following aspects:   - Comparative added value. - For devices with equivalent value or added value, the evaluation proceeds to economic evaluation and price negotiation. | (50–52) |
| **Slovakia** | | **Reimbursement of MD and IVD under institutional care.** | HTA is not a formal part of any reimbursement pathway. However, it may be used for certain medical devices.  HTA assessments are only used to support decision-making; they don’t constitute the decision. | The HTA considers the following aspects:   - Health problems and current use of technology. - Technical characteristics. - Clinical effectiveness and safety. - Economic assessment. - Ethical, organizational, social, patient and legal aspects. | (53–56) |
| **Spain** | | **Reimbursement of medical devices in the inpatient setting** (under the common Benefit Package)**.** | HTA by RedETS is a preliminary mandate for updating the content of the common package.  HTA recommendations are the basis of reimbursement decisions. | The HTA and reimbursement decisions consider the following aspects:   - Comparative effectiveness. - Safety. - Cost-effectiveness, budget, organizational, social, and ethical impacts. | (57,58) |
| **Sweden** | | **National reimbursement of medical devices based on the joint cooperation model for assessing and procuring medical technology**. | HTA is conducted (by TLV) for selected technologies identified by the Medical Technology Product Council.  However, reimbursement decisions are regional and not necessarily based on HTA. | The HTA recommendations consider the following aspects:   - Clinical evidence. - Health economic analysis. | (59–61) |
| **UK** | **England** | **Reimbursement of medical devices by NHS England.** | HTA by NICE is a formal part of reimbursement pathways for selected technologies.  Reimbursement decisions by NHS England are primarily based on NICE guidance; however, only technology appraisal guidance is legally binding. | HTA recommendations consider the following aspects:   - Clinical benefits, including comparative effectiveness outcomes. - Cost analysis.   Reimbursement decisions by NHS England consider the following aspects:   - NICE guidance recommendations. - The cost impact of the technology on NHS England.^****^ | (62–64) |
|  | **Scotland** | **Reimbursement of medical devices by NHS Scotland.** | HTA is not a formal part of any reimbursement pathway. However, SHTG may conduct HTA for certain devices on an ad-hoc basis.  NHS Scotland is required to consider the HTA recommendations in their reimbursement decisions if available. | HTA recommendations consider the following aspects:   - Comparative effectiveness. - Safety. - Cost-effectiveness. - Patients’ and professionals’ perspectives. - Broader impact on the healthcare system. | (65,66) |
|  | **Wales** | **Reimbursement of medical devices by NHS Wales.** | HTA is not a formal part of any reimbursement pathway. However, HTW may conduct it for certain devices on an ad-hoc basis.  NHS Wales is expected to follow HTW guidance or recommendations if available.  Also, NICE’s Technology Appraisal Guidance is legally binding in Wales. | HTA recommendations consider the following aspects:   - Comparative effectiveness. - Cost-effectiveness. - Other considerations (including equity, environmental sustainability, or other priority issues in Wales). | (67,68) |
| **Norway** | | **Reimbursement of new medical devices under the managed introduction of new technologies in the specialist health care service (Nye metoder).** | HTA is a formal part of “**Nye metoder”** where selected medical devices are assessed by NOMA through a national HTA.  The national-level assessments are used to inform decision-makers who may also take into account other factors. | HTA recommendations consider the following aspects:   - Potential benefits - The device’s impact on the healthcare system. - Associated risks. - Budgetary implications.   The reimbursement decisions consider three unique aspects of the Norwegian context:   - The benefit criteria. - The resource criteria. - The severity criteria. | (69,70) |

**LKF:** The Austrian procedure and diagnosis-oriented groups. **AIHTA:** Austrian Institute for Health Technology Assessment. **CTIIMH-CRIDMI**: The Commission of Reimbursement of Implants and Invasive Medical Devices. **CTM:** Medical Technical Council. **CHIF:** Croatian Health Insurance Fund. **MoH:** Ministry of Health. **DHTC:** Danish Health Technology Council. **EHIF**: Estonian Health Insurance Fund. **FinCCHTA:** Finnish Coordinating Center for Health Technology Assessment. **DRG:** Diagnosis Related Group. **CNEDiMTS:** Medical Device and Health Technology Evaluation Committee. **HAS:** French National Authority for Health. **InEK:** Hospital Remuneration System. **G-BA**: Federal Joint Committee in Germany. **SHI:** Statutory Health Insurance in Germany. **IQWiG**: The Institute for Quality and Efficiency in Healthcare. **TéF:** Technology Assessment Department in Hungary. **VASPVT**: State Accreditation Service for Health Care Activities under the Ministry of Health of the Republic of Lithuania. **ZIN:** the National Health Care Institute in the Netherlands. **AOTMiT:** Agency for Health Technology Assessment and Tarification. **CATS:** The Portuguese Commission for Health Technology Assessment. **RedETS:** Spanish Network of Health Technology Assessment Agencies. **TLV:** The Dental and Pharmaceutical Benefits Agency. **NICE:** National Institute for Health and Care Excellence (England). **HTW:** Health Technology Wales. **SHTG**: Scottish Health Technologies Group. **NHS**: National Health Service (UK). **NOMA:** Norwegian Medical Products Agency (previously called the Norwegian Medicines Agency).

* The nine categories are (1) Intracranial stents used in angioplasty of atheromatous stenosis, (2) Conventional implantable cardiac defibrillators with endocardial lead (single-, dual-, and triple-chamber), (3) Implantable cardiac defibrillators without endocardial lead (single-, dual-, and triple-chamber), (4) Biological surgical heart valves, (5) Implantable devices for the treatment of pelvic organ prolapse by the vaginal route, (6) Implantable devices for the treatment of urinary incontinence by the vaginal route, (7) Devices for the treatment of pelvic organ prolapse by the abdominal route, (8) Intracranial flow diverter stents (9) Thrombectomy devices.

** The IQWiG conducts the HTA on behalf of the G-BA.

*** Class IIb devices fall under this rule only if they work by emitting energy.

^****^ For medical technology guidance, funding by NHS England requires that the technology has positive NICE guidance, cost-saving within 3 years of implementation (demonstrated through NICE modelling) and is affordable to the NHS (the cost should not exceed £20 million in any of the first 3 years).

^†^ In the Netherlands, the reimbursement system for MD/IVD represents an open system, as the technologies can be reimbursed without a formal HTA assessment. However, the National Health Care Institute (Zorginstituut Nederland; ZIN) acts as a referee in cases of disagreement between physicians, patients, and health insurance companies when there is uncertainty about whether a new treatment qualifies for reimbursement under the Health Care Package or in case of uncertainty about whether it is sufficiently effective, particularly when a new treatment is involved. In such cases, ZIN assesses the technology and issues an HTA assessment with a recommendation. Health insurers may only reimburse such treatment under basic insurance if ZIN has issued a positive assessment outcome.

***Potential mechanisms to address uncertainty regarding safety and effectiveness for high-risk MD/IVD***

Detailed information on each country’s mechanisms and requirements for evidence uncertainty are presented in Supplementary Table S11.

**Supplementary Table S12**. Mechanisms to address the uncertainty of comparative safety and effectiveness evidence for MD/IVD in selected countries

| Country | Mechanism(s) to address uncertainty | Criteria for eligibility and/or prioritization | Summary of the process and reimbursement conditions | Ref. |
| --- | --- | --- | --- | --- |
| Austria | **1. Recommended with restriction** after HTA assessment by AIHTA. | In case of evidence uncertainty, but the evidence suggests the possibility of additional benefit. | - The intervention (including the device) will be included as a fully reimbursable service but coupled with certain limitations (for example, it will only be available in selected hospitals with specific qualifications). - The service might be subject to reassessment later after completion of registered, ongoing randomized clinical trials or fulfilling other conditions. | (6,7) |
|  | **2. Preliminary exclusion** after HTA assessment by AIHTA | In case the evidence of the clinical benefit is inconclusive or unavailable, and long-term effectiveness and safety are uncertain. | - The intervention is not reimbursed, but it may still be included in the hospital individual services catalogue as a new examination and treatment method (receive the XN-Code), allowing a three- to five-year monitoring and re-evaluation. - Updated assessments can be later conducted, leading to either (a) an inclusion as a reimbursable benefit, (b) prolongation of the preliminary status, or (c) the non-inclusion of medical service. |  |
| Belgium | **1. Category G (restricted clinical application; RCA) in the list** | In case there is uncertainty about the added value of a medical device compared with existing therapeutic alternatives. | - The decision includes restricted reimbursement conditions (specify healthcare centres or providers’ requirements and the indications for which reimbursement is approved). - The reimbursement decision includes a defined period for both reimbursement and re-evaluation. - The inclusion may also limit the number of devices reimbursed annually or in total, and criteria must be defined for inclusion on a nominative list. | (8–14) |
| Denmark | **1. Recommendation for use in knowledge acquisition after assessment by DHTC.** | If the available data suggests that **(a)** the technology may be significantly better than existing alternatives, but more key data is needed about its safety and cost-effectiveness, or **(b)** the technology may be highly cost-effective compared with existing alternatives, but there is insufficient evidence that it is as effective or safe as the comparator. | - This recommendation can include a recommendation for not using the technology in the health system until the results of the ongoing knowledge-acquisition process are available, a recommendation to use the technology until the Council reassesses the recommendation based on the results of the knowledge-acquisition process or no recommendation in favour or against using the technology during the knowledge-acquisition stage (status quo). - The reimbursement decision may also impose restrictions on location, specifying that the technology will be used at one or a few selected hospitals, and concurrently with this use, more knowledge will be collected to address insufficiencies. - The company is responsible for covering the costs of the knowledge acquisition phase. However, the Council may recommend that the five regional governments cover the expenses in exceptional cases. | (19) |
| France | **1. Temporary coverage under Article L165-1-5 of the French Social Security Code.** | For CE-marked innovative devices that are intended to treat serious or rare diseases or to address unmet medical needs. | - The devices are assessed for eligibility by CNEDiMTS. - Reimbursement decisions for this scheme are made by the Ministers of Health and of Social Security following CNEDiMTS’s Evaluation. - The scheme offers one-year temporary reimbursements pending conventional reimbursement via the LPPR. | (23,24,71) |
|  | **2. Innovation Funding (IF) scheme.** | For innovative medical devices, with limited early evidence but, are liable to provide a substantial benefit to health or reduce health expenditures. | - The applicant submits a proposal with a full draft protocol of the proposed study to HAS and to the Ministries of Health and of Social Security. - HAS assesses the application’s eligibility based on the type of technology, the technology’s innovative character, and the relevance of the clinical or health economic results. - Once HAS completes its review, it issues an opinion on the request, which could be one of three outcomes: recommended, not recommended, or recommended with observation. | (23,24,72,73) |
| Germany | **1.** **G-BA initiated clinical studies.** | If neither benefit, harm, nor ineffectiveness can be identified by the IQWiG assessment of a device, the G-BA can either wait for clinical studies (ongoing elsewhere) or initiate a clinical study in Germany. | - IQWiG develops the study’s fundamental design (PICO question, study design (RCT in most cases), study duration/length of follow-up, secondary outcomes, etc). - The study’s funding will be provided by G-BA unless the manufacturer offers to finance the trial. | (32,73) |
|  | **2. Coverage with the evidence development scheme (CED) (§137e SGB V).** | In case of evidence uncertainty, the CED scheme allow for the reimbursement of devices while additional evidence is being collected.  The device must meet the criteria of “potential” (that is, promising effectiveness). | - A dossier is submitted for CED by manufacturers and those who apply the new procedures. - The G-BA makes the final decision on incorporation in the CED after the IQWiG assessment. - The study itself is again funded by the G-BA. - Once the study is completed, the G-BA arrives at a final conclusion on general reimbursement. |  |
| The Netherlands | **Potentially Promising Care Pathway** | For innovative medical devices and services with uncertainty in the long-term value. | - Enables conditional reimbursement of devices strictly within research conditions. Devices approved under this scheme are not added to the basic healthcare package. Instead, funding is contingent on conducting parallel research to collect data on the device’s safety, effectiveness, and cost-effectiveness during the reimbursement period. - The program is currently closed to new applications; discussions are ongoing regarding the possibility of initiating a new iteration or reviving the program. | (43,73,74) |
| Spain | **Post-launch evidence-generation studies (Estudios de Monitorización)** | Quantitative prioritization criteria are used for coverage decisions. The criteria include disease burden, innovation element, safety, financial and organizational aspects, costs and other implications. | - Technologies are identified by the National Commission of Provision, Insurance and Financing (CPAF) of the Ministry of Health and selected by the Directorate General of the common portfolio of services of the National Health System (NHS) and Pharmacy (DGPSPh), which are usually identified from previous HTA reports from RedETS. - Devices selected for coverage are reimbursed only with research in selected healthcare centres identified at the regional level. | (75) |
| England | **1. Guidance recommendation for use only in Research after NICE assessment** | For technology with insufficient or uncertain evidence but with potential benefits. | - The recommendation comes without a requirement for NHS funding. The device is only recommended for use exclusively in research settings. | (64) |
|  | **2. Guidance recommendation with data collection** | For technologies that could provide substantial benefits to patients or healthcare systems. However, considerable uncertainty exists about whether the expected benefits are realizable in the NHS. | - The device may be fully or partially used as an option in the NHS during the evidence-generation period. |  |

**AIHTA:** Austrian Institute for Health Technology Assessment. **DHTC:** Danish Health Technology Council. **EHIF:** Estonian Health Insurance Fund. **CNEDiMTS:** Medical Device and Health Technology Evaluation Committee. **HAS:** French National Authority for Health. **G-BA:** Federal Joint Committee in Germany. **IQWiG:** The Institute for Quality and Efficiency in Healthcare. **RCT:** Randomised Controlled Trial. **PICO**: patient/population, intervention, comparison and outcomes. **RedETS:** Spanish Network of Health Technology Assessment Agencies. **NICE:** National Institute for Health and Care Excellence. **NHS:** National Health Service (UK).

## Regional variations in the reimbursement framework and decisions implementation

The level of centralization in the reimbursement framework for each country is described in detail in Supplementary Table S13.

**Supplementary Table S13.** Regional variations in the reimbursement frameworks for high-risk medical devices in the EU/EEA/UK as of November 2024.

| Country | Centralization of reimbursement frameworks | Description of expected regional variations |
| --- | --- | --- |
| Austria | Decentralized/ Mixed | The implementation of reimbursement decisions (final purchasing decisions) is handled at the hospital level or by centralized public hospital cooperations in the federal states.  The nine federal states manage healthcare delivery separately in the inpatient sector. |
| Belgium | Generally centralized | The Minister of Social Affairs and Public Health nationally implements the decision formulated by the CTIIMH-CRIDMI with budgetary approval from the Minister of Budget.  However, hospitals can cover the cost of certain medical devices or procedures even when they are not officially reimbursed, as the national position on reimbursement is not always available when new devices enter the Belgian market. |
| Croatia | Generally centralized | National HTA reports are centrally processed by Croatian decision-makers. |
| Denmark | Decentralized / Mixed | The recommendations from the DHTC are not legally binding for the regional governments, but they are expected to follow them unless they have special reasons to deviate from them. The regional governments’ procurement organizations and the Regions Joint Procurement are responsible for the procurement and tendering procedures. |
| Estonia | Generally centralized | The EHIF oversees and funds medical services, including devices, through a national list of healthcare services. Based on EHIF evaluation and decisions, necessary changes in regulations (positive list of medical devices, positive list of health services, etc) are made by the Ministry of Social Affairs. |
| Finland | Generally centralized | FinCCHTA reports are generated jointly by the five university hospitals, resulting in national-level adaptation of technology. Also, after COHERE Finland’s positive recommendations, Kela reimburses the technology nationally, resulting in generally centralized reimbursement.  However, rapid HTA can be conducted at the ‘WellBeing Services County’ level for specific high-risk medical devices to support local reimbursement decisions. |
| France | Generally centralized | Generally, the French healthcare system is highly centralized, as reimbursement under DRG or LPPR after CNEDiMTS assessments has a centralized implementation. However, hospitals can still evaluate and procure medical devices through different local evaluation (hospital-based HTA) and reimbursement systems. |
| Germany | Generally centralized | Generally, G-BA decisions are centrally implemented. However, regional variations are still expected, especially in the NUB scheme, as regional or hospital-by-hospital approaches are used to price and reimburse hospital medical devices. |
| Hungary | Generally centralized | The National Health Insurance Fund decides on reimbursement after consulting the National Centre for Public Health and Pharmacy (NNGYK) and the Health Professions College. The decisions are centrally implemented.  There are no regional differences in the reimbursement process for drugs or medical devices. |
| Italy | Decentralized/ Mixed | Patient’s access to medical devices is arranged separately in different Italian regions. Unlike medicinal products, where reimbursement approval and procurement processes are centralized and strictly regulated, decision-making in relation to the purchasing of medical devices is left to individual hospital committees.  The pricing of some devices (for example, knee prostheses and coronary stents) is decided nationally using reference prices. However, each region can adjust the price to suit local healthcare needs. |
| Lithuania | Generally centralized | Generally, the VASPVT assesses medical devices, and the recommendations are assessed and implemented centrally by the Minister of Health and VLK, respectively. |
| Netherlands | Generally centralized | The assessment and decision-making are generally centralized. The HTA is conducted by ZIN and the Minister of Health, Welfare and Sport to determine whether to include or exclude medical devices in the basic package. |
| Poland | Generally centralized | The Minister of Health centrally decides on reimbursement and reimbursement conditions after AOTMiT’s assessment.  However, decisions by AOTMiT may not necessarily be implemented throughout Poland, resulting in regional variation in technology reimbursement. |
| Portugal | Generally centralized | INFARMED generally develop national recommendations. The Minister of Health decides on the recommendations for approval and national reimbursement. |
| Spain | Decentralized/ Mixed | Medical devices can be assessed and procured independently in the 17 autonomous Spanish regions, with the authority to make decisions differently through regional executive and legislative mechanisms.  Devices can also be assessed nationally by RedETS before being reimbursed nationally by the Common Portfolio of Services of the Spanish NHS. |
| Sweden | Decentralized/ Mixed | Each of Sweden’s 21 regions and 290 municipalities handles the provision and procurement of MD within their respective healthcare budgets.  However, selected medical devices may undergo a national-level assessment by TLV, and the MTP Council makes reimbursement decisions for these selected devices through the joint cooperation model for assessing and procuring medical technology. |
| UK | Decentralized/ Mixed | HTA is conducted separately at three different HTA organizations: NICE in England, SHTG in Scotland, and HTW in Wales. Reimbursement decisions are made in each of the four UK nations: NHS England, NHS Scotland, NHS Wales and Health and Social Care in Northern Ireland.  There is no formal legal requirement for jurisdictions within the UK to adopt HTA recommendations or reimbursement decisions from other regions, with the exception of NICE’s Technology Appraisal Guidance (TAG), which is legally binding in Wales. However, HTA recommendations from different UK regions may be considered on an ad hoc basis to inform local or hospital-level procurement decisions. |
| Norway | Generally centralized | The system for managed introduction of new technologies in the specialist health care service (Nye metoder) is responsible for the national introduction of new technologies. The national HTA is conducted by NOMA, and final decisions are made by the Decision Forum.  The Decision Forum is a decision-making group in the national system consisting of the chief executive officers of the four Regional Health Authorities.^*^ |

**CTIIMH-CRIDMI**: Commission of Reimbursement of Implants and Invasive Medical Devices. **MoH**: Ministry of Health. **DHTC**: Danish Health Technology Council. **EHIF**: Estonian Health Insurance Fund. **FinCCHTA**: Finnish Coordinating Center for Health Technology Assessment. **COHERE Finland**: Council for Choices in Health Care in Finland. **Kela**: Social Insurance Institution of Finland. **DRG**: Diagnosis-Related Group. **LPPR**: List of Products and Services Reimbursable by Health Insurance (France). **CNEDiMTS**: Medical Device and Health Technology Evaluation Committee (France). **G-BA**: Federal Joint Committee (Germany). **NUB**: New Examination and Treatment Methods (Germany). **NNGYK**: National Centre for Public Health and Pharmacy (Hungary). **VASPVT**: State Accreditation Service for Health Care Activities (Lithuania). **VLK**: National Health Insurance Fund (Lithuania). **ZIN**: National Health Care Institute (Netherlands). **AOTMiT**: Agency for Health Technology Assessment and Tarification (Poland). **INFARMED**: National Authority for Medicament and Health Products (Portugal). **RedETS**: Spanish Network of Health Technology Assessment Agencies. **NHS**: National Health Service. **NICE**: National Institute for Health and Care Excellence (England). **SHTG**: Scottish Health Technologies Group. **HTW**: Health Technology Wales. **TAG**: Technology Appraisal Guidance (produced by NICE). **MTP Council**: Medical Technology Product Council (Sweden). **TLV**: Dental and Pharmaceutical Benefits Agency (Sweden). **NOMA**: Norwegian Medical Products Agency. **Nye metoder**: The Norwegian National System for Managed Introduction of New Health Technologies. **Decision Forum**: Norwegian national decision-making group comprising the chief executive officers of the four Regional Health Authorities

* The four RHAs in Norway are: Central Norway Regional Health Authority (“Helse Midt-Norge”); Northern Norway Regional Health Authority (“Helse Nord”); Southern and Eastern Norway Regional Health Authority (“Helse Sør-Øst”); and Western Norway Regional Health Authority (“Helse Vest”).

# References

1. Estonian Health Insurance Fund. Medical devices [Internet]. [cited 2024 Sep 9]. Available from: https://www.tervisekassa.ee/en/people/medical-devices

2. Estonian Health Insurance Fund. List of Health Care Services and General Information on Amending the List [Internet]. [cited 2024 Sep 9]. Available from: https://www.tervisekassa.ee/en/partner/list-health-care-services

3. Josipović I. DECISION PROMULGATING THE MEDICAL DEVICES ACT I hereby promulgate the Medical Devices Act passed by the Croatian Parliament at its session on 14 June 2013. The President of the Republic of Croatia. 2013.

4. Rulebook on the procedure for placing orthopedic and other aids on the basic and additional list of orthopedic and other aids and determining the prices of orthopedic and other aids [Internet]. ; 2019 [cited 2024 Dec 27]. Available from: https://narodne-novine.nn.hr/clanci/sluzbeni/2019_07_69_1438.html

5. Huic M, Tandara Hacek R, Svajger I. HEALTH TECHNOLOGY ASSESSMENT in CENTRAL, EASTERN, and SOUTH EUROPEAN COUNTRIES: CROATIA. Int J Technol Assess Health Care. 2017;33(3):376–83.

6. FAQ | Austrian Medical Devices Registry [Internet]. [cited 2025 Jan 2]. Available from: https://medizinprodukteregister.at/FAQs#toc-are-medical-devices-reimbursed-in-austria-in-the-out-patient-sector-in-the-in-patient-sector-and-how-does-it-work

7. Goetz G, Panteli D, Busse R, Wild C. Reimbursement decisions for medical services in Austria: an analysis of influencing factors for the hospital individual services catalogue between 2008 and 2020. BMC Health Serv Res [Internet]. 2022 Dec 1 [cited 2025 Jan 2];22(1):1–13. Available from: https://bmchealthservres.biomedcentral.com/articles/10.1186/s12913-022-07531-3

8. Justel databank [Internet]. [cited 2024 Dec 27]. Available from: https://www.ejustice.just.fgov.be/cgi_loi/change_lg.pl?language=fr&la=F&cn=2014062503&table_name=loi

9. Reimbursement of implants and invasive medical devices | RIZIV [Internet]. [cited 2024 Dec 27]. Available from: https://www.riziv.fgov.be/nl/professionals/individuele-zorgverleners/verstrekkers-van-implantaten/terugbetaling-implantaten-en-invasieve-medische-hulpmiddelen

10. Reimbursement of implants and invasive medical devices | INAMI [Internet]. [cited 2024 Dec 27]. Available from: https://www.riziv.fgov.be/fr/professionnels/professionnels-de-la-sante/fournisseurs-d-implants/remboursement-des-implants-et-des-dispositifs-medicaux-invasifs#.WilO_a2WyUk

11. Baeyens H, Pouppez C, Slegers P, Vinck I, Hulstaert F, Neyt M. Towards a guided and phased introduction of high-risk medical devices in Belgium [Internet]. 2015 [cited 2024 Dec 27]. Available from: https://kce.fgov.be/en/publications/all-reports/towards-a-guided-and-phased-introduction-of-high-risk-medical-devices-in-belgium

12. Van Voorde C DE, Van Den Heede K, Obyn C, Quentin W, Geissler A, Wittenbecher F, et al. Conceptual framework for the reform of the Belgian hospital payment system [Internet]. 2014 [cited 2024 Aug 25]. Available from: https://kce.fgov.be/en/publications/all-reports/conceptual-framework-for-the-reform-of-the-belgian-hospital-payment-system

13. Camberlin C, Obyn C, Neyt M. Transversal Budget Impact Analysis [Internet]. 2023 [cited 2024 Dec 27]. Available from: https://kce.fgov.be/en/publications/all-reports/transversal-budget-impact-analysis

14. In vitro diagnostic medical devices (IVD): reimbursement request | INAMI [Internet]. [cited 2025 Jan 6]. Available from: https://www.inami.fgov.be/fr/professionnels/autres-professionnels/fabricants-et-distributeurs-d-implants-de-div-et-d-autres-dispositifs-medicaux/dispositifs-medicaux-de-diagnostic-in-vitro-div-demande-de-remboursement

15. Agency for Quality and Accreditation in Health Care, Croatia The Croatian Guideline for Health Technology Assessment Process and Reporting [Internet]. 2011. Available from: www.aaz.hr

16. General Health Insurance Company of the Czech Republic (VZP) [Internet]. [cited 2025 Feb 24]. Joint methodologies of General Health Insurance Company of the Czech Republic (VZP CR) and Association of Health Insurance Companies of the Czech Republic (SZP CR) for uncategorized ZP. Available from: https://www.vzp.cz/poskytovatele/ciselniky/zdravotnicke-prostredky/spolecne-metodiky-vzp-cr-a-szp-cr-pro-nekategorizovane-zp

17. General Health Insurance Company of the Czech Republic (VZP) [Internet]. [cited 2025 Feb 24]. Inclusion, change and increase in the price and reimbursement of ZUM in the Reimbursement Catalogue of VZP – ZP . Available from: https://www.vzp.cz/poskytovatele/ciselniky/zdravotnicke-prostredky/zarazeni-zmena-a-navyseni-ceny-a-uhrady-zum-v-uhradovem-katalogu-vzp-zp

18. The Danish Health Technology Council’s methods guide for the evaluation of health technology. 2023.

19. The Danish Health Technology Council’s process guide 2021. 2021.

20. Oulu University Hospital [Internet]. [cited 2024 Sep 9]. National Assessment Network. Finnish Coordinating Center for Health Technology Assessment (FinCCHTA). Available from: https://oys.fi/fincchta/kansallinen-arviointiverkosto/

21. Oulu University Hospital [Internet]. [cited 2024 Sep 9]. Proceedings. Finnish Coordinating Center for Health Technology Assessment (FinCCHTA). Available from: https://oys.fi/fincchta/katsauksia-ja-suosituksia/

22. Council for Choices in Health Care in Finland (COHERE Finland) [Internet]. [cited 2024 Dec 29]. Council for Choices in Health Care in Finland (COHERE Finland). Available from: https://palveluvalikoima.fi/en/frontpage

23. Haute Autorité de Santé - National Committee for the Evaluation of Medical Devices and Health Technologies (CNEDiMTS*) [Internet]. [cited 2024 Dec 27]. Available from: https://www.has-sante.fr/jcms/c_2036238/en/national-committee-for-the-evaluation-of-medical-devices-and-health-technologies-cnedimts?portal=p_3058934&userLang=en

24. Pathway of medical devices in France [Internet]. 2017 Nov [cited 2024 Aug 21]. Available from: https://www.has-sante.fr/jcms/p_3308477/en/guide-pathway-of-medical-devices-in-france.

25. Omelyanovsky V V., Otstavnov SS, Musina NZ, Dombrovskii VS. Introduction of medical products into healthcare practice and costs reimbursement: European experience. FARMAKOEKONOMIKA Modern Pharmacoeconomics and Pharmacoepidemiology [Internet]. 2018 Aug 15 [cited 2024 Dec 27];11(2):59–66. Available from: https://www.pharmacoeconomics.ru/jour/article/view/252

26. Ghabri S, Josselin JM, Le Maux B. Could or Should We Use MCDA in the French HTA Process? Pharmacoeconomics [Internet]. 2019 Dec 1 [cited 2024 Dec 27];37(12):1417–9. Available from: https://link.springer.com/article/10.1007/s40273-019-00846-w

27. Pesqué R, Percheron R, Cordonnier AL, Steelandt J, Paubel P, Pineau J, et al. Accès au marché des dispositifs médicaux innovants : articulation entre évaluations nationales et hospitalières (Health technology assessment of innovative medical devices: Timing and decision at national and local level). Ann Pharm Fr. 2020 Mar 1;78(2):189–97.

28. Institute for Quality and Efficiency in Health Care (IQWiG) [Internet]. [cited 2024 Aug 24]. When and how does IQWiG assess non-drug interventions? Available from: https://www.iqwig.de/en/presse/in-the-focus/assessment-of-non-drug-interventions/2-when-and-how-does-iqwig-assess-non-drug-interventions/

29. Beck ACC, Retèl VP, Bhairosing PA, van den Brekel MWM, van Harten WH. Barriers and facilitators of patient access to medical devices in Europe: A systematic literature review. Health Policy (New York). 2019 Dec 1;123(12):1185–98.

30. Busse R, Blümel M, Knieps F, Bärnighausen T. Statutory health insurance in Germany: a health system shaped by 135 years of solidarity, self-governance, and competition. The Lancet [Internet]. 2017 Aug 26 [cited 2024 Dec 30];390(10097):882–97. Available from: http://www.thelancet.com/article/S0140673617312801/fulltext

31. Olberg B, Fuchs S, Matthias K, Nolting A, Perleth M, Busse R. Evidence-based decision-making for diagnostic and therapeutic methods: The changing landscape of assessment approaches in Germany. Health Res Policy Syst [Internet]. 2017 Oct 17 [cited 2024 Dec 30];15(1):1–11. Available from: https://health-policy-systems.biomedcentral.com/articles/10.1186/s12961-017-0253-1

32. Institute for Quality and Efficiency in Health Care (IQWiG) [Internet]. [cited 2024 Dec 30]. Assessments according to §137h SGB V . Available from: https://www.iqwig.de/en/about-us/methods/results/assessments-according-to-137h/

33. The Federal Ministry of Justice [Internet]. [cited 2024 Dec 30]. Ordinance on the requirements for the assessment of new examination and treatment methods with high-risk medical devices according to Section 137h of the Fifth Book of the Social Code (Medical Device Methods Assessment Ordinance - MeMBV). Available from: https://www.gesetze-im-internet.de/membv/__2.html

34. Federal Joint Committee (Gemeinsamer Bundesausschuss - G-BA) [Internet]. [cited 2024 Aug 24]. The benefit assessment of medicinal products in accordance with the German Social Code, Book Five (SGB V). Available from: https://www.g-ba.de/english/benefitassessment/

35. 28/2010. (V. 12.) EüM Decree on the professional criteria and policy priorities to be applied in the procedure related to the inclusion of health technologies used in curative and preventive procedures in health insurance financing [Internet]. 2010 [cited 2025 Jan 15]. Available from: https://net.jogtar.hu/jogszabaly?docid=a1000028.eum

36. HSE website - Health Service Executive - HSE.ie [Internet]. [cited 2025 Jan 13]. Available from: https://www.hse.ie/

37. DECREE-LAW 19 June 2015, n. 78 [Internet]. 2015 [cited 2025 Feb 20]. Available from: https://www.normattiva.it/uri-res/N2Ls?urn:nir:stato:decreto.legge:2015-06-19;78!vig

38. Tarricone R, Amatucci F, Armeni P, Banks H, Borsoi L, Callea G, et al. Establishing a national HTA program for medical devices in Italy: Overhauling a fragmented system to ensure value and equal access to new medical technologies. Health Policy (New York). 2021 May 1;125(5):602–8.

39. The State Accreditation Service for Health Care Activities under the Ministry of Health. [Internet]. [cited 2024 Dec 23]. Health technology assessment. Available from: https://techmed.vaspvt.gov.lt/sveikatos-technologij%C5%B3-vertinimas/sveikatos-technologij%C5%B3-vertinimas/

40. Lithuanian Parliament. Order of the Minister of Health of the Republic of Lithuania No. V-208 on the procedure for reimbursement of medical devices [Internet]. 2016 Jan [cited 2024 Dec 29]. Available from: https://www.e-tar.lt/portal/lt/legalAct/f1c0e290c01e11e5a6588fb85a3cc84b/asr

41. Lithuanian Parliament. Order on the Approval of the Description of the Procedure for the Evaluation of Applications for Contracts from Orthopaedic Undertakings and Economic Operators and for the Publication of Information on the Submission and Evaluation of Such Applications [Internet]. 2019 Feb [cited 2024 Dec 29]. Available from: https://www.e-tar.lt/portal/lt/legalAct/cb4a957036a411e99595d005d42b863e/asr

42. Package management in practice 4: Package management as solid basis for appropriate care. 2023 Mar.

43. Assessment State of Science and Practice 2023 [Internet]. 2023 Apr [cited 2024 Sep 29]. Available from: https://english.zorginstituutnederland.nl/publications/reports/2019/04/23/assessment-of-established-medical-science-and-medical-practice%E2%80%99---a-technical-modification

44. Advising on and clarifying the contents of the standard health care benefit package | About us | National Health Care Institute [Internet]. [cited 2025 Jan 29]. Available from: https://english.zorginstituutnederland.nl/about-us/working-methods-and-procedures/advising-on-and-clarifying-the-contents-of-the-standard-health-care-benefit-package

45. Koperny M, Konieczna M, Głowik P, Siwiec J, Dziurda D, Sejbuk K, et al. Health Technology Assessment Guidelines for Medical Devices. 2021.

46. Medical devices assessment guidelines [Internet]. [cited 2024 Sep 16]. Available from: https://www.aotm.gov.pl/en/guidelines/medical-devices-assessment-guidelines/

47. Tajstra M, Dyrbus M, Grabowski M, Rokicki JK, Nowak M, Gasior M. The use of remote monitoring of patients with cardiac implantable electronic devices in Poland. Polish Heart Journal (Kardiologia Polska) [Internet]. 2022 [cited 2024 Dec 29];80(4):479–81. Available from: https://journals.viamedica.pl/polish_heart_journal/article/view/KP.a2022.0050/66691

48. Health technology assessment guidelines. Medical devices. Products for prophylactic and therapeutic use PART I [Internet]. Warsaw; 2021 Jun [cited 2024 Dec 29]. Available from: https://www.aotm.gov.pl/wp-content/uploads/2022/04/Wytyczne-Oceny-Technologii-Medycznych.-Wyroby-medyczne-czesc-I.pdf

49. Agency for Health Technology Assessment and Tariff System (AOTMiT) [Internet]. [cited 2024 Sep 16]. Assessment of reimbursement applications. Available from: https://www.aotm.gov.pl/en/medicines/assessment-of-reimbursement-applications/

50. Vinhas J, Dias S, Gouveia AM, Correia A, Dias C V, Sousa D, et al. Pharmacotherapeutic assessment methodology, Version 3.0. Committee for Health Technology Assessment [Internet]. 2023 [cited 2024 Sep 19]. Available from: https://www.infarmed.pt/documents/15786/1963929/METOD_AFT_v3.0_ENvf_fev2023/b0cb1c54-adca-721a-6466-75ba04cdd542

51. HTA Country/Area Profile (Portugal) [Internet]. 2020 [cited 2024 Sep 19]. Available from: https://cdn.who.int/media/docs/default-source/health-economics/hta-country-profiles-2020-21/hta-country_area-profile_portugal.pdf?sfvrsn=720b2741_3

52. Caldeira S, Santos C, Furtado C, Cossito M. PORTUGAL. PPRI Conference. Vienna; 2019.

53. Act of 13 September 2011 on the scope and conditions of reimbursement of medicines, medical devices and dietetic foods under public health insurance and on amendment and supplementation of certain acts [Internet]. [cited 2025 Jan 7]. Available from: https://www.slov-lex.sk/ezbierky/pravne-predpisy/SK/ZZ/2011/363/#poznamky.poznamka-1ca

54. Announcement of the Ministry of Health of the Slovak Republic for manufacturers of medical devices that will be subject to the categorization of special medical materials [Internet]. [cited 2025 Jan 7]. Available from: https://www.health.gov.sk/Clanok?oznam-kk-szm-20121009

55. National Institute for Health Outcomes (NIHO) [Internet]. 2021 [cited 2024 Aug 19]. How does the system work in Slovakia? Available from: https://niho.sk/ako-funguje-system-na-slovensku/

56. Notification procedure of medical devices in Slovakia | ŠÚKL [Internet]. [cited 2024 Dec 30]. Available from: https://www.sukl.sk/en/medical-devices/instructions/registration-notification-procedure-of-medical-devices-in-slovakia?page_id=1718

57. EUnetHTA WP7 research and analysis activity 1: Final report An analysis of HTA and reimbursement procedures in EUnetHTA partner countries: final report.

58. Serrano-Aguilar P, Asua-Batarrita J, Molina-López MT, Espallargues M, Pons-Rafols J, García-Armesto S, et al. The Spanish Network of Agencies for Health Technology Assessment and Services of the National Health System (RedETS). Int J Technol Assess Health Care [Internet]. 2019 [cited 2025 Jan 13];35(3):176–80. Available from: https://pubmed.ncbi.nlm.nih.gov/31006412/

59. The MTP Council [Internet]. [cited 2024 Sep 23]. National Joint Introduction - Collaborative medicine technology. Available from: https://samverkanmedicinteknik.se/ordnat-inforande/national-joint-introduction

60. Dental and pharmaceutical benefits agency (TLV) [Internet]. [cited 2024 Sep 23]. Nationally ordered introduction of medical technology products - Dental and pharmaceutical benefits agency TLV. Available from: https://www.tlv.se/medicinteknikforetag/nationellt-ordnat-inforande-av-medicintekniska-produkter.html

61. Dental and pharmaceutical benefits agency (TLV) [Internet]. 2024 [cited 2024 Sep 23]. HTA regulation - Dental and pharmaceutical benefits agency TLV. Available from: https://www.tlv.se/medicinteknikforetag/hta-forordningen.html

62. National Health Service [Internet]. [cited 2025 Jan 31]. Understanding routes to NICE health technology assessment - AI and Digital Regulations Service for health and social care. Available from: https://www.digitalregulations.innovation.nhs.uk/regulations-and-guidance-for-developers/all-developers-guidance/understanding-routes-to-nice-health-technology-assessment/

63. Diagnostics Assessment Programme manual [Internet]. 2011 Dec [cited 2025 Jan 31]. Available from: https://www.nice.org.uk/Media/Default/About/what-we-do/NICE-guidance/NICE-diagnostics-guidance/Diagnostics-assessment-programme-manual.pdf

64. NICE health technology evaluations: the manual [Internet]. 2023 Oct [cited 2024 Aug 19]. Available from: https://www.nice.org.uk/guidance/pmg36/resources/nice-health-technology-evaluations-the-manual-pdf-72286779244741

65. A mechanism for the consideration of SHTG national advice on health technologies: a guide for NHS Boards [Internet]. 2024 [cited 2025 Jan 29]. Available from: https://shtg.scot/media/2443/20230623-consideration-of-national-advice-on-health-technologies_a-guide-for-boards-v20.pdf

66. Scottish Health Technology Group Products. 2022 Aug.

67. Appraisal Process Guide - Health Technology Wales [Internet]. 2024 Aug [cited 2025 Jan 27]. Available from: https://healthtechnology.wales/wp-content/uploads/Appraisal-Process-Guide.pdf

68. Health Technology Wales [Internet]. [cited 2024 Oct 30]. Our Appraisal Process - Health Technology Wales. Available from: https://healthtechnology.wales/about/our-appraisal-process/

69. Guidance criteria for management of medical devices in the National System for Managed Introduction of New Health Technologies within the Specialist Health Service in Norway (Nye metoder) [Internet]. 2018 Jan [cited 2024 Dec 29]. Available from: https://www.nyemetoder.no/48ff64/siteassets/documents/om-systemet/guidance-criteria-for-handling-medical-devices-in-nye-metoder.pdf

70. Odd HA, Helga F. PPRI Pharma Profile Norway [Internet]. 2022 [cited 2024 Aug 23]. Available from: https://ppri.goeg.at/system/files/inline-files/PPRINorway2022_final.pdf

71. Innovation funding: submission of an application for exceptional funding for an innovative product [Internet]. 2015 Apr [cited 2024 Dec 12]. Available from: https://www.has-sante.fr/jcms/p_3238091/fr/submission-of-an-application-for-exceptionnal-funding-for-an-innovative-product

72. Innovation funding: submission of an application for exceptional funding for an innovative product [Internet]. Available from: www.has-sante.fr

73. Federici C, Reckers-Droog V, Ciani O, Dams F, Grigore B, Kaló Z, et al. Coverage with evidence development schemes for medical devices in Europe: characteristics and challenges. Eur J Health Econ [Internet]. 2021 Nov 1 [cited 2024 Dec 11];22(8):1253–73. Available from: https://pubmed.ncbi.nlm.nih.gov/34117987/

74. Care Innovation Stories: An exploration of four innovation examples in healthcare [Internet]. 2021 Jun [cited 2024 Sep 25]. Available from: https://www.zorginstituutnederland.nl/publicaties/rapport/2021/06/25/kpmg-rapport-zorginnovatieverhalen

75. Serrano-Aguilar P, Gutierrez-Ibarluzea I, Díaz P, Imaz-Iglesia I, González-Enríquez J, Castro JL, et al. Postlaunch evidence-generation studies for medical devices in Spain: the RedETS approach to integrate real-world evidence into decision making. Int J Technol Assess Health Care [Internet]. 2021 [cited 2024 Dec 30];37(1):e63. Available from: https://www.cambridge.org/core/journals/international-journal-of-technology-assessment-in-health-care/article/postlaunch-evidencegeneration-studies-for-medical-devices-in-spain-the-redets-approach-to-integrate-realworld-evidence-into-decision-making/A705E811A6EEF5865100588156A95E7C

# 
